# Quantum Confinement Emissions in Strained Monolayer WSe_2_: A Nanoscale Approach to Single-Photon Emitters via Tip-Enhanced Techniques

**DOI:** 10.1021/acsnano.5c18642

**Published:** 2026-03-21

**Authors:** Lucas Liberal, Rafael Battistella Nadas, Gustavo H. R. Soares, Frederico B. Sousa, Maria Clara Godinho, Gabriel Marques Jacobsen, Takashi Taniguchi, Kenji Watanabe, Marcio Daldin Teodoro, Ado Jorio, Leonardo Cristiano Campos

**Affiliations:** † Departamento de Física, 28114 Universidade Federal de Minas Gerais, Belo Horizonte, Minas Gerais 31270-901, Brazil; ‡ Departamento de Física, 67828 Universidade Federal de São Carlos, São Carlos, SP 13565-905, Brazil; § Institut für Physik, Humboldt-Universität zu Berlin, Newtonstraße 15, 12489 Berlin, Germany; ∥ Institut für Experimentelle und Angewandte Physik, Universität Regensburg, 93053 Regensburg, Germany; ⊥ Research Center for Materials Nanoarchitectonics, 52747 NIMS, Tsukuba 305-0044, Japan; # Research Center for Electronic and Optical Materials, NIMS, Tsukuba 305-0044, Japan; ∇ Centro de Tecnologia em Nanomateriais e Grafeno, Belo Horizonte, Minas Gerais 31310-260, Brazil

**Keywords:** NanoPL, strain engineering, single-photon emitters, two-dimensional materials, nanomaterials, TMDs

## Abstract

Two-dimensional (2D) semiconductors such as monolayer WSe_2_ have attracted significant interest for their quantum properties and potential as scalable single-photon emitters. However, conventional microphotoluminescence (μ-PL) techniques are fundamentally limited by optical diffraction, hindering access to critical nanoscale features such as strain gradients and localized quantum confinement. In this study, we utilize tip-enhanced photoluminescence (NanoPL) with a spatial resolution of ≈10 nm to directly image the emission landscape of monolayer WSe_2_ on top of nanopillars at room temperature. Our results reveal two distinct localization regimes associated with leading theoretical models for single-photon activation and provide guidelines for deterministic nanoengineering of quantum light sources.

## Graphical abstract

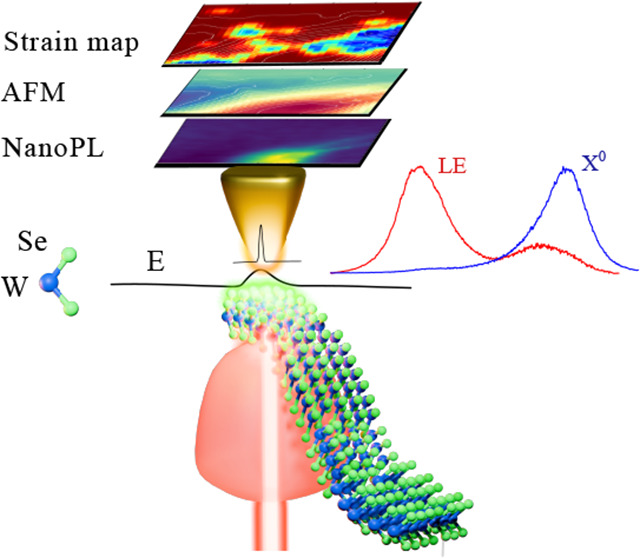

## Introduction

Transition metal dichalcogenides (TMDs) in their monolayer form, such as tungsten diselenide (WSe_2_), have emerged as a versatile platform for investigating unique quantum phenomena including spin-valley lock,^1–4^ many-body effects^5–8^ and the development of single-photon sources for controlled light manipulation.^9–16^ Their intrinsically two-dimensional nature, coupled with strong Coulomb interactions and large exciton binding energies,^1,17^ give rise to robust optical properties that are highly sensitive to external perturbations such as strain,^18–21^ defects,^1,22,23^ and variations in the dielectric environment.^24,25^ In particular, the controlled application of strain through engineered nanostructures, such as nanopillars, has proven to be an effective strategy for nanopositioning localized excitonic states that act as single-photon emitters (SPEs),^9–15^ a critical component for future quantum technologies. However, despite notable advances, a comprehensive understanding of the spatial and spectral characteristics of these localized emissions at the nanoscale remains limited, primarily due to the resolution constraints of conventional optical techniques.

Microphotoluminescence (μ-PL) spectroscopy, although extensively used to investigate monolayer TMDs, is fundamentally limited by the optical diffraction limit, which restricts spatial resolution to several hundred nanometers.^26^ Even with high numerical aperture (NA) objectives or oil-immersion techniques, the minimum resolvable distance typically exceeds 400 nm,^27^ making μ-PL limited for investigating nanoscale structures such as nanopillars, which commonly exhibit dimensions on the order of 150 nm.^9,13^ More precisely, μ-PL intrinsically averages the signal over the laser spot area, thus concealing critical nanoscale variations and local phenomena, such as strain gradients,^20,28,29^ quantum confinement effects,^30,31^ and defect-related emissions.^22,32^ Despite the growing number of experimental observations, the physical origin of SPE in TMDs remains under debate. A precise understanding of the strain field distribution is essential to delineate the boundaries between competing theoretical models. One prevailing hypothesis attributes SPE formation to defect-assisted brightening of dark exciton states,^33^ a mechanism consistent with observations of the *g*-factor, valley dynamics, and the natural occurrence of SPEs near the flake boundary. However, recent studies have shown the confinement of lower-energy states near the edges of TMD monolayer nanobubbles^30,31,34^ can result from nonuniform strain and strain pockets which aligns with alternative confinement mechanisms that do not require the presence of atomic-scale defects. These overlapping signatures and nanoscale origin complicate the task of isolating the dominant process responsible for SPE activation, especially when both mechanisms may coexist within the same nanostructure.

While several techniques—such as tip-enhanced photoluminescence have been employed to probe nanoscale emission,^35–38^ and even to access dark excitons at room temperature,^39^ direct and unambiguous measurements of spectral features induced by nanopillars remain elusive. In this work, we address this gap by providing a detailed nanoscale mapping of the photoluminescence response of individual nanopillars at room temperature. By combining bottom-illuminated photoluminescence measurements with a plasmonically tunable scanning probe tip, we achieve spatial resolution down to 10 nm, enabling the visualization of quantum confinement emissions within the nanopillar region. We systematically investigate distinct nanopillar geometries, correlating strain profiles with low energy localized emissions to identify distinct confinement regimes compatible with different theoretical descriptions. Our results reveal the coexistence of two main localization mechanisms: (i) strain-induced potential confinement, characterized by emission extended across the full nanopillar area with spatial variations preserved over 100 nm; and (ii) defect-hybridized confinement, which produces highly localized emission spots down to ≈10 nm, closely matching the resolution limit of the technique. These two regimes exhibit distinct spatial and spectral signatures, underscoring the need to consider both mechanisms when interpreting SPE behavior in 2D materials. Furthermore, the analysis of distorted pillars with irregular geometries allows us to visualize exciton diffusion over extended distances and to probe the spatial propagation of strain fields induced by the nanopillar. Altogether, our work provides a high-resolution framework for studying exciton diffusion and confinement regimes in single-photon emitters samples, establishing a foundation for optimal nanoengineering of quantum light sources in two-dimensional semiconductors.

## Results and Discussion

In our NanoPL experiments, we employed the Porto-SNOM laboratory prototype,^40^ equipped with an oil-immersion objective lens (NA = 1.4) and a radially polarized HeNe laser (632.8 nm) operating at 1.9 μW of laser power at the sample. Plasmon-tunable Tip Pyramids^41^ were used as probe tips. In this setup, the laser beam passes through the glass coverslip, the nanopillar structure, and the WSe_2_ monolayer before interacting with the metallic tip (see schematics in Figure 1a). The backscattered PL signal is collected along the same optical path and directed to the detector. The combination of a high-NA lens and tip-enhancement allowed us to collect high-intensity spectra even at low excitation power, which is ideal for studying defect-related and SPE. These typically saturate at excitation powers around 3.0 μW (as studied in previous work^42^). The NanoPL collection is performed concomitant with an atomic force microscopy (AFM) measurement, enabling a direct correlation between the PL signal and topography. All NanoPL colormaps are obtained by subtracting the far-field (FF) signal from the near-field (NF) signal. In the backscattering configuration, the tip-down measurement (tip engaged) contains both NF and FF contributions, while the tip-up measurement (tip retracted by 1 μm) probes only the FF background. Both maps are acquired using identical experimental parameters, and the NF component is isolated by subtracting the tip-up from the tip-down signal. The Supporting Information is available online and includes a dedicated section (“Far-field/Near-field Subtraction”) providing a detailed description of this procedure, as well as additional discussions of the strain calculation methods.

**Figure 1:**
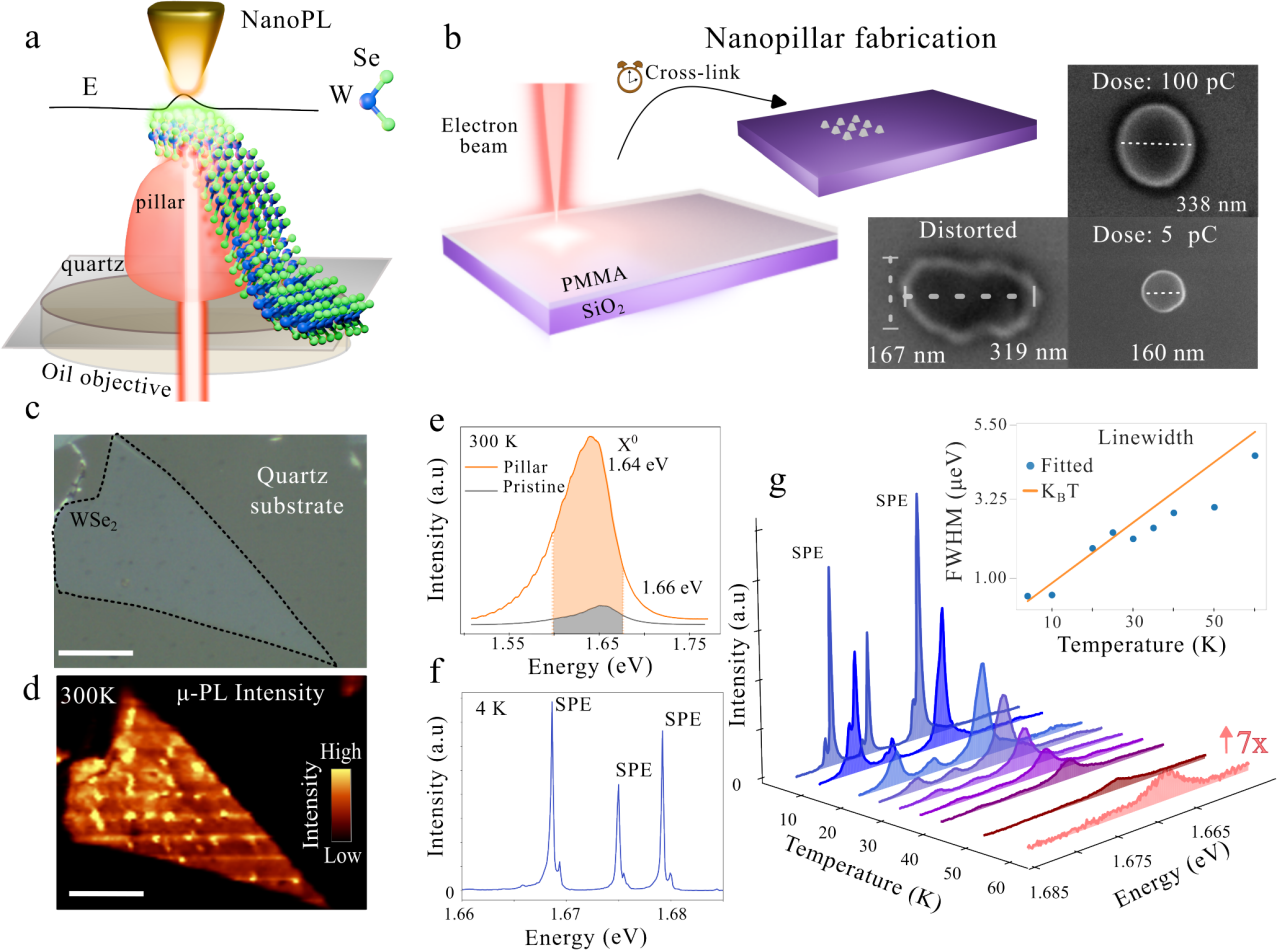
SPE sample characterization (a) Schematic of the NanoPL experimental setup. The gold tip approaches from the top, while the laser is directed from below, passing through an oil-immersion objective (NA = 1.4) and the transparent nanopillar before reaching the WSe_2_. (b) Nanopillar fabrication process. Nanopillars were defined in SiO_2_ using PMMA and high electron-beam lithography doses, which determine their final dimensions. Inadequate corrections of aperture and astigmatism, along with charge accumulation effects from the substrate, can lead to distorted pillar shapes. (c) Optical image of WSe_2_ deformed by nanopillars on a quartz substrate. (d) μ-PL intensity map integrated over the energy interval displayed in (e), measured at room temperature comparing the nanopillar signal with flat TMD. The scale bar is 10 μm. (e) Room-temperature μ-PL spectra illustrating exciton funneling toward the nanopillars, with an 8× enhancement in quantum yield and a redshift. (f) Spectrum at *T* = 4 K showing three high-intensity SPEs with narrow line widths (fwhm ≈ 400 μeV). (g) Temperature dependence of a single-photon emitter, highlighting line width broadening and quenching as temperature increases. The inset compares line width broadening with *k*_B_T. Figures (e) and (f) are reproduced with permission from ref 42. Copyright 2025 Royal Society of Chemistry.

With this setup in mind, SPE samples were developed in WSe_2_ using transparent nanopillars to ensure light transmission through the nanostructure. For this purpose, high electron beam lithography doses were employed - corresponding to long dwell times - which induced cross-linking in the PMMA (poly­(methyl methacrylate)) resist, rendering it into a rigid structure, as shown in Figure 1b. By carefully tuning the exposure dose, aperture settings, and correcting for astigmatism, well-defined, circular structures are reproducibly produced with diameters as small as 150 nm. Moreover, inadequate adjustments and charge accumulation effects induced by the substrate can result in distorted nanopillars, as illustrated in Figure 1b. These effects will be further discussed in later sections of the manuscript. The transmission NanoPL setup, combined with transparent nanopillars, provides favorable conditions for NanoPL studies on nanopillar-based samples by significantly suppressing background contributions and minimizing additional optical signals typically associated with metallic nanopillars and reflective substrates. This experimental configuration facilitates the investigation not only of wrinkles and nanobubbles,^34,39,43^ but also of nanopillar-induced strain profiles, which remain largely unexplored in NanoPL experiments. The sample preparation with the specifics sample variations is described in the Experimental Section (Sample Information).

An optical image of the WSe_2_ monolayer deposited on a quartz substrate is shown in Figure 1c. The corresponding μ-PL intensity map, recorded at room temperature under an excitation power of 100 μW (Figure 1d), reveals a pronounced exciton μ-funneling effect directed toward the nanopillars. For comparison, the reference spectrum without nanopillars is presented in Figure 1e. To confirm single-photon emission, μ-PL measurements were performed at 3.6 K under an excitation power of 1 μW using another sample with the same nanopillar geometry but in a SiO_2_ substrate, with the TMD encapsulated in hBN.^42^ High-quality SPE spectra were obtained, exhibiting filtered signals, strong intensities, narrow line width and typical saturation behavior, as shown in Figure 1f and detailed in previous work.^42^ Importantly, it has also been demonstrated that these nanostructures enable the observation of dark doublets associated with SPE arising from emissions localized at the curved sidewalls of the nanopillars.^42^ Therefore, the ability to probe the nanopillar at the nanometric scale is essential for understanding and optimizing SPE design.

Temperature dependence of the SPE is illustrated in Figure 1g, emphasizing the quenching effect of thermal energy and the increase in line width on the localized emissions. It was already showed that by creating deep defects is possible to observe the SPE emission up to *T* = 150 K, far beyond the upper limit of 70 K for our experiments.^10^ However, this limit is only applied for the μ–PL. The first NanoPL hyperspectral map at room temperature is presented in Figure 2 with a 300 × 300 nm square scan dimension with pixel size of ≈10 nm and revealing three distinct regions with varying strain profiles and corresponding PL responses. Our analysis focuses on a flat region (exhibiting the standard WSe_2_ emission), a wrinkle, and the border of the nanopillar, each region highlighted in the nanoPL intensity map (integrated from 1.50 to 1.60 eV) and corroborated by AFM measurements (see Figure 2a,b). Specific local PL spectra are shown in Figure 2c. A clear red shift in the bright exciton (*X* ^0^) position is observed in both the wrinkle and nanopillar regions when compared with the flat region in blue (standard WSe_2_ emission) due to strain introduction^18,21,44^ (35 meV for the wrinkle and 22 meV for the nanopillar border). Even though the wrinkle has a higher shift in the *X* ^0^, translating in higher strain, the nanopillar presents a more intense and lower energy peak at 1.530 eV which is attributed to a localized emission (LE), highlighted in the NanoPL intensity map of Figure 2a. This emission is distant from the *X* ^0^ by 117 meV, excluding the possibility of being a solely trion or dark exciton induced by the near field regime, since the energy difference is incompatible (dark exciton usually with 30 to 60 meV^43,45^ from *X* ^0^ and trions on the order of 30 meV^46,47^ ). In the FF measurement, no localized emissions is detected, confirming the need of the NF spectroscopy given the strong spatial confinement of the emission (See SI Figure 2).

**Figure 2:**
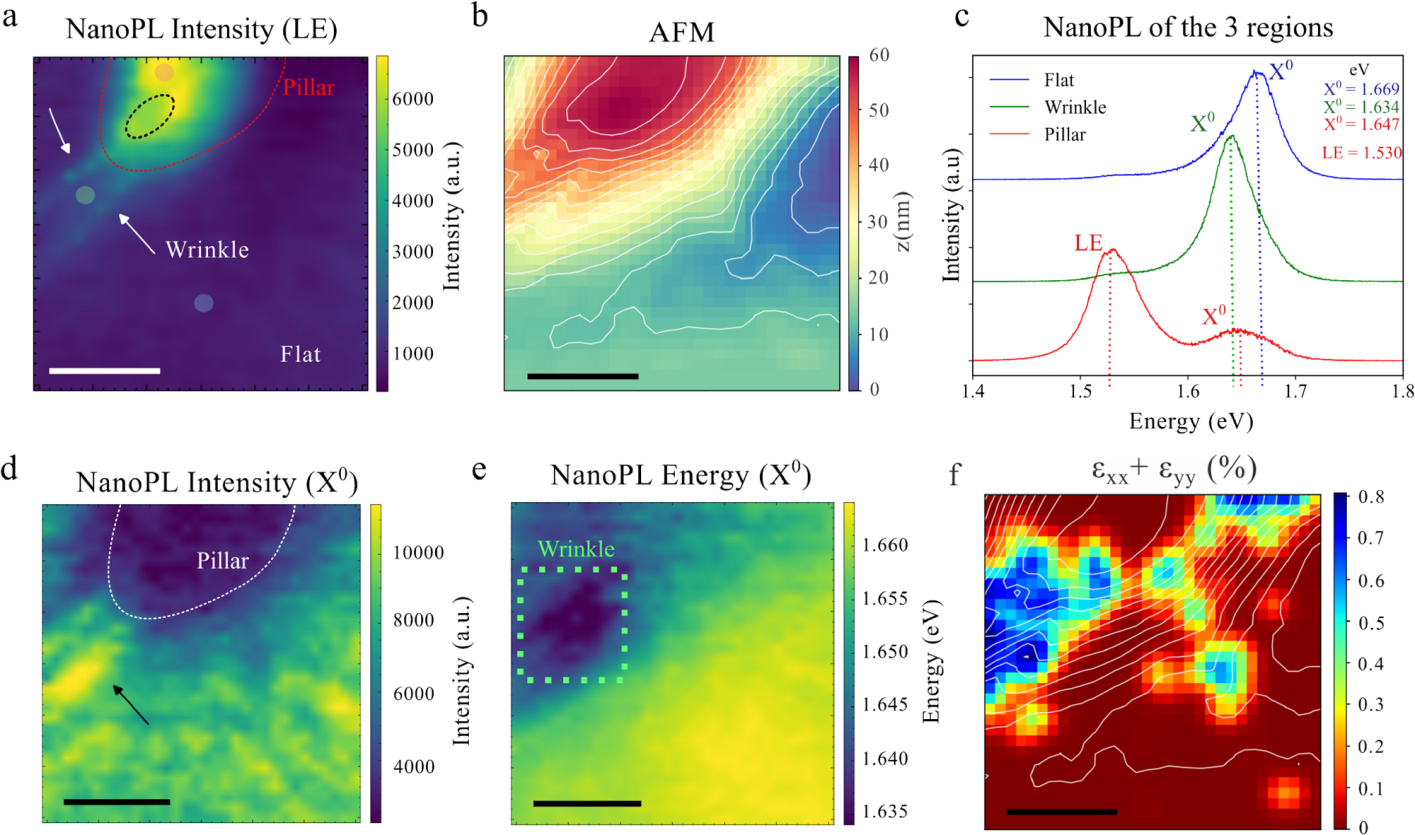
NanoPL map of a 300 × 300 nm region showing nanopillar borders and wrinkles. (a) NanoPL intensity map of the localized emission (LE), which appears exclusively within the nanopillar region. The red contour marks the nanopillar boundary; the black area corresponds to a local height maximum indicating the onset of a wrinkle; white arrows indicate wrinkles induced by the nanopillar structure. Outside the nanopillar, the TMD remains flat. The faded dots represent the PL spectra showed in (c). (b) AFM topography of the same region. The white contour lines represent height variations of 4.2 nm. (c) NanoPL spectra from three selected regions (displayed in (a) with faded dot with corresponding colors), showing distinct photoluminescence behaviors and the energy shift between the LE and *X* ^0^. (d) NanoPL intensity map of the *X* ^0^ emission, which is enhanced in the wrinkle region and suppressed within the nanopillar. (e) Energy map of the *X* ^0^ emission. (f) Strain tensor colormap (ϵ_*xx*_ + ϵ_*yy*_) of the nanopillar, indicating enhanced strain localized at the wrinkle. All scale bars correspond to 100 nm.

To address the apparent contradiction between the presence of a strong low-energy (LE) emission and the comparatively smaller strain inferred from the reduced *X* ^0^ energy shift at the nanopillar region relative to the wrinkle, we estimated the strain profile by calculating the strain tensor using the method described in ref 48. This approach extracts the local strain components ϵ_*xx*_ and ϵ_*yy*_ directly from AFM topography by analyzing surface curvature through second-order spatial derivatives, while incorporating the Poisson ratio and appropriate boundary conditions. A detailed description of the method, along with a discussion of its inherent limitations, is provided in the Supporting Information (Section Strain Calculation) and in ref 48. Figure 2f shows the distribution of the trace of the strain tensor for studied topography. A relatively high strain, reaching approximately 0.8%, is observed within the wrinkle region, consistent with the measured redshift of about 35 meV assuming uniaxial strain.^49–51^ In contrast, at the location of the strongest LE emission, the calculated strain is close to zero. This discrepancy reflects intrinsic limitations of curvature-based continuum models,^30,48^ which are primarily sensitive to sharp local variations in topography, as highlighted by the white contour lines in Figure 2b,f. Importantly, the redshift of the *X* ^0^ emission in this region indicates that strain is indeed present, even if its magnitude is underestimated by the strain extraction method. Both the strain tensor calculation and the NanoPL maps consistently indicate higher strain in the wrinkle region, which is reasonable given its quasi-one-dimensional geometry. Beyond the strain magnitude itself, the intensity behavior reveals a more complex excitonic dynamics. While both the wrinkle and nanopillar regions exhibit signatures of exciton funneling (see SI Figure 2d for the total integrated nanoPL spectra), the dominant emission differs: in the wrinkle, funneling primarily enhances the *X* ^0^ emission, whereas in the nanopillar region it predominantly amplifies the LE emission. This highlights the limitations of a simplified interpretation based solely on far-field μ-PL (as depicted in Figure 1d,e), where strain effects manifest mainly as a broad, asymmetric red-shifted peak without spatial resolution.

Remarkably, the LE intensity in the nanopillar region becomes comparable to the *X* ^0^ emission observed in the wrinkle, which is unusual for a emission arising purely from a strongly localized state. Two main mechanisms may contribute to this behavior and explain the high energy distance of 117 meV presented. First, strain-induced hybridization between dark excitons and defect levels can generate low-energy LE states, similar to the mechanism commonly invoked to explain SPE formation at cryogenic temperatures.^33,44^ This interpretation is supported by our low-temperature measurements, suggesting that strain-induced hybrid states play a significant role which can be identified even at room temperature.^44^ Second, strain can locally modify the atomic structure, creating nanoscale potential wells that confine excitons and lead to multiple low-energy emission sites near nanopillar edges through nanowrinkling effects, as reported in refs 30,31,34 . These highly localized strain pockets caused by nonuniform strain are inaccessible to continuum theory^30,48^ and would not be visible within our strain tensor calculus. While both mechanisms are consistent with SPE behavior at low temperatures, they differ in the degree of exciton localization at room temperature and the need for defects, and it remains unclear how the spatial distribution of these emissions evolves as the temperature decreases. Given that both effects occur at the nanoscale, experimentally disentangling them remains challenging. It is therefore likely that the observed LE emission results from a combined contribution of strain-induced confinement and defect-related hybridization. Further investigation of the power dependence of the LE emission was not feasible, as the TMD structure on the nanopillar collapsed when the excitation power was increased to 19 μW (see SI Figure 4). Although this power is relatively low for room-temperature PL measurements, the early collapse suggests strong local field enhancement provided by the top at the nanopillar, further supporting the presence of efficient exciton funneling toward this region.

To gain deeper insight into the LE, we now examine the upper region of the same nanopillar, where measurements were carried out over a small scan area of 80 × 80 nm^2^ (Figure 3a–c) with pixel size of 2.5 nm. Figure 3a,b display the LE spatial and energy distribution, revealing that the emission is markedly inhomogeneous across the nanopillar surface. The strongest signal emerges from a confined zone—approximately matching the tip diameter (≈10 nm)—located away from the apex, and the LE peak exhibits a noticeable blue shift as it approaches the center of the pillar (as displayed in Figure 3e). This observation is consistent with theoretical predictions attributing maximum strain and exciton confinement to the periphery of strain-induced nanobubbles.^30^ In Figure 3e, a series of spectra acquired along a vertical path - closely aligned with the height gradient toward the nanopillar apex- showcase the LE energy evolution and its abrupt intensity quenching. Simultaneously, the *X* ^0^ exhibits a concurrent increase in brightness without a significant energy shift, suggesting distinct strain responses in energy and a intensity competition between the two emission channels (a more detailed analyses of the *X* ^0^ is compromised due to the FF subtraction from the NF and the proximity of the strained *X* ^0^ and the pristine *X* ^0^). Notably, the LE is strongly position dependent, with its energy shifting by up to 17 meV across the 80 nm span, accompanied by a reduction of 70% of the peak intensity. Although higher strain is indicated in the lower part of the colormap, neither the AFM topography nor the calculated strain tensor (Figure 3c,d) can account for the strong spatial localization of the LE emission. Figure 3f presents a schematic representation of the approximate position of the analyzed region within the symmetric nanopillar shown in Figure 2. Within this framework, the LE emission appears to be localized toward the nanopillar periphery and progressively weakens as it approaches the apex. The calculated strain map for the symmetric nanopillar, shown in SI Figure 1, reveals localized strained regions whose spatial distribution and strain magnitudes are consistent with those observed in Figure 3.

**Figure 3:**
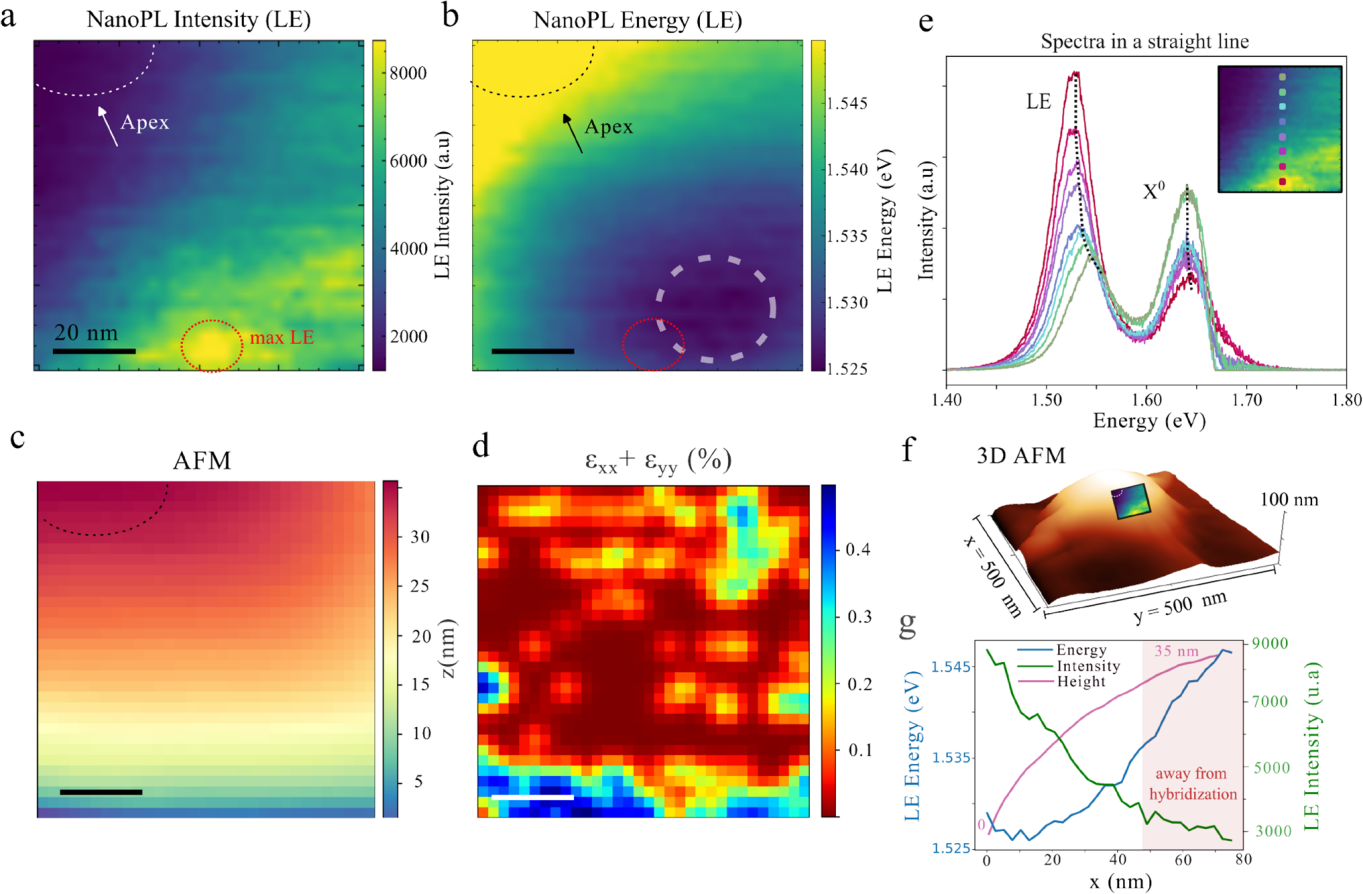
Pillar apex behavior of the localized emission (LE). (a) NanoPL intensity map (80 × 80 nm^2^) of the LE. The white dotted arc marks the nanopillar apex, while the red dotted arc outlines the region of maximum LE intensity. (b) NanoPL energy map of the LE. The gray region indicates the area of minimum emission energy. (c) AFM topography of the same region. (d) Strain tensor colormap (ϵ_*xx*_ + ϵ_*yy*_). (e) NanoPL spectra along a vertical line showing the quenching of LE emission toward the nanopillar apex. (f) 3D plot of the AFM for the entire nanopillar. The respective region of the NanoPL studied is illustrated in the AFM. (g) LE energy, intensity and height plotted together along the same vertical axis. For higher energy regions, a strong quenching effect is observed, with the emission reduced to 30% of its initial intensity. All scale bars correspond to 20 nm.

Figure 3g compares the energy shift and intensity suppression of LE with the AFM topography, revealing a strong localization and correlation between lower-energy states and brighter emission, consistent with charge funneling and exciton confinement toward local energy minima. Nevertheless, as illustrated by the gray circular segment in Figure 3b which depicts the minimum energy zone, the emission near 1.525 eV extends beyond the area of maximum LE intensity, which is laterally displaced as highlighted in red in Figure 3a. This spatial mismatch suggests that charge funneling alone cannot fully explain the localization, pointing to an additional mechanism. A plausible explanation involves defect-assisted hybridization, as emphasized in Figure 3g and consistent with reports showing that dark-exciton hybridization spans a finite energy range, with maximum intensity occurring near defect-related energy levels.^44^ Furthermore, SI Figure 3 presents data along a horizontal scan line (80 nm) with minimal topographic variation (<1 nm), where a 12 meV energy shift and a >50% drop in emission are still observed underscoring that such extreme spatial localization (<10 nm) is unlikely attributed only to topographical or strain. Although strong localization could, in principle, arise from nanowrinkling not captured by the strain colormaps, additional support for a defect-related mechanism comes from the next nanopillar, which exhibits a markedly different strain configuration yet shows two distinct localized emission peaks, one of which occurs at the same energy (≈1.53 eV) as the LE state discussed in Figure 3.

### Distorted Pillars

Up to this point, the studied nanopillars exhibited a well-rounded shape with a height of 100 nm and a diameter of approximately 170 nm, corresponding to an aspect ratio of 1.17 (height/radius). These dimensions significantly exceed the theoretical models for strain-induced states,^30,31,34^ and are comparable to those typically used in SPE studies.^9,52^ The nanopillars were initially fabricated on SiO_2_ substrates and subsequently transferred to quartz^53^ to ensure optical transparency for nanoPL. To simplify the fabrication process, an alternative approach involved patterning the nanopillars directly onto the quartz substrate. However, this change led to charge accumulation effects due to the poor electronic conductivity of quartz. Consequently, deformed nanopillars—such as the one shown in Figure 1b—were frequently observed. These structures exhibited pronounced anisotropy, with the lateral dimensions along the *x* and *y* directions being significantly different and both exceeding the 85 nm radius of the structures analyzed in the previous section. The morphological deformation resulted in altered PL responses and a much lower aspect ratio, closer to those predicted in theoretical models for localized electronic states.^30,31,34^

The first example of the nanoPL response from a distorted nanopillar is shown in Figure 4 with 1 × 1 μm with a step size of 16 nm. For this structure, three distinct emission features are observed. The *X* ^0^ is present throughout the entire mapping region, with its energy and intensity modulated by the topographic features of the pillar (colormaps for the *X* ^0^ behavior is available in the SI Figure 6). In addition, two lower-energy peaks appear within the nanopillar region (Figure 4a,b), each exhibiting distinct characteristics. The most intense among them, labeled *X*_s_, spans the entire nanopillar area and exhibits an energy offset of approximately 60 meV from the *X* ^0^ (Figure 4e), which is consistent with the expected value for dark exciton states.^1,8,39^ Although the *X*_s_ emission is activated by the strain induced by the pillar, it extends well beyond the pillar region, with detectable intensity up to a maximum of 400 nm away from the structure, also appearing in the FF spectra, in high contrast from the LE (see SI Figure 5c,d for FF and NF comparison). This presence outside the nanopillar weakens the possibility of a dark exciton enhancement with due to the tip proximity.^39,54^ Its maximum intensity occurs in a specific peripheral region (Figure 4c,d), which spatially coincides with two distinct apexes that act as nearly orthogonal topography variations (see 3D AFM in SI Figure 5). Despite having a well-defined maximum, the *X*_s_ emission is broadly distributed spatially, retaining more than 50% of its maximum intensity over a 400 nm range (Figure 4f)—markedly contrasting with the highly localized LE emission in Figure 3 or in Figure 4b. The calculated strain map shows maximum strain in between the left and right corners of the distorted nanopillar, partially correlating with the LE emission but only on one side. Interestingly, the strain magnitude is nearly twice that of the symmetric nanopillar presented in Figure 3, even though the LE emission occurs at similar energy with comparable intensity and confinement. The strain distribution, however, extends over a much larger area than the highly localized emission. This asymmetry is compatible with additional mechanisms, such as defect-assisted carrier confinement, which likely contribute to a significantly larger magnitude of the LE energy shift and its spatial distribution compared to the calculated strain in Figure 4d. In contrast, the region dominated by *X*_s_ shows almost no calculated strain, which appears inconsistent given the nanopillar’s topographic inhomogeneities and multiple axes. Notably, the model is limited in capturing shear strain ϵ_*xy*_, leading to underestimation of strain values and potentially missing local variations due to the continuum approximation and the 16 nm AFM step size. However, the strong *X*_s_ intensity and lower energy combined with the NanoPL energy map for the *X* ^0^ showed in SI Figure 6b resembles the behavior expected from potential wells, which can give rise to lower-energy emission spread over extended regions in lower aspect-ratio regimes.^30,31^ Another important observation is the pronounced quenching of the *X* ^0^ emission (black spectrum in Figure 4e), suggesting a more efficient relaxation into the *X*_s_ state compared to the LE observed in the previous nanopillar. This behavior may be related to the energetic proximity between the *X*_s_ and dark and bright states, which could facilitate enhanced *X*_s_ emission or related to saturation effects due to the defect nature of the LE. Notably, this trend is not observed in subsequent results involving deeper *X*_s_ energies, where such intensity relation is more subtle. The resulting *X*_s_/*X* ^0^ intensity ratio reaches 7.7, more than twice the value of 3.6 measured in the nanopillar of Figure 3 for the LE/*X* ^0^.

**Figure 4:**
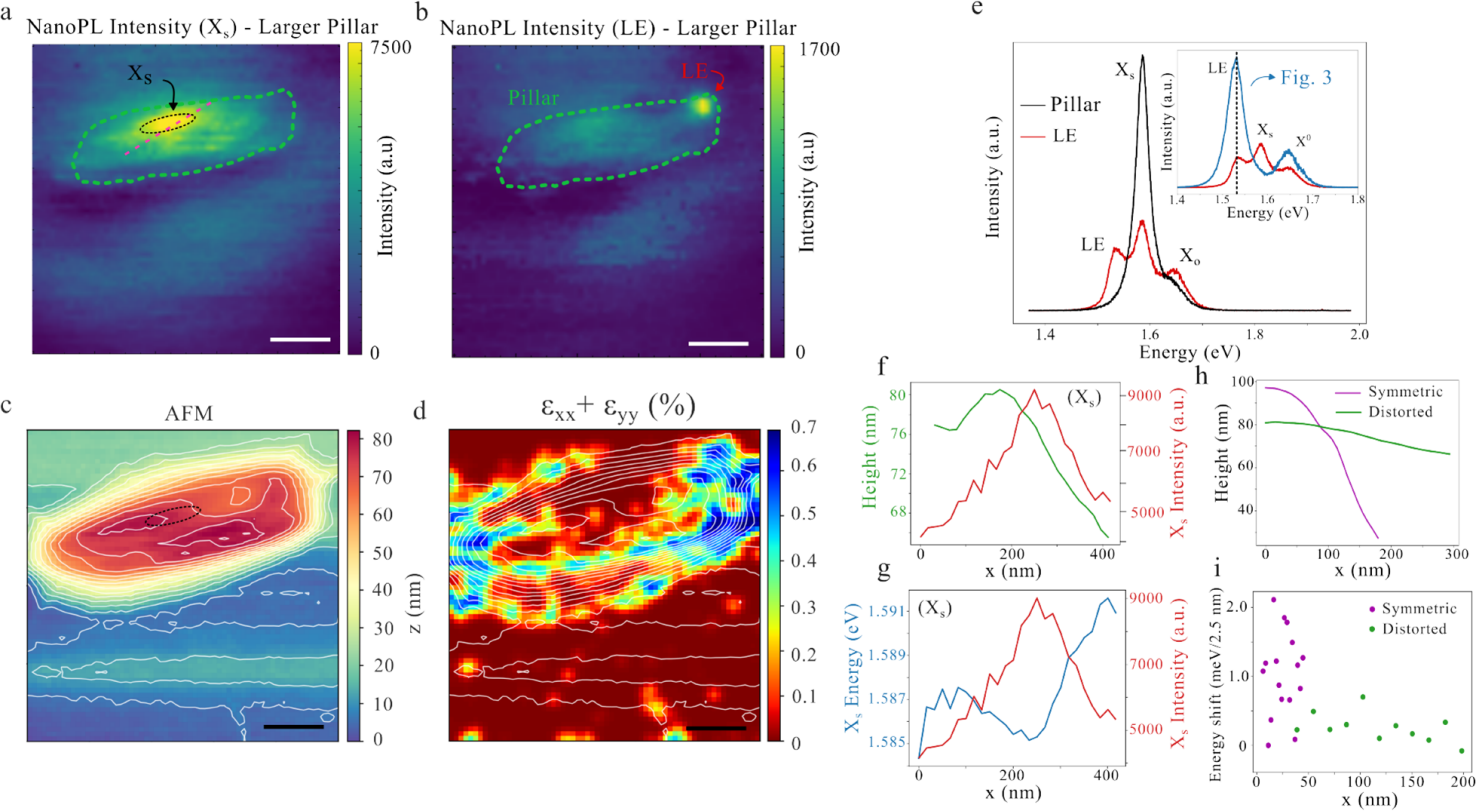
Distorted nanopillar 1 – multiple apexes. (a) NanoPL intensity colormap of the *X*_s_ emission, showing a maximum near two local height maxima (apexes), with strong emission across the nanopillar and reduced intensity outside. (b) NanoPL intensity map of the LE emission, highly localized and confined to an area of 3 × 3 pixel. (c) AFM topography of the nanopillar, highlighting two local maxima. The white contour lines represent height variations of 5.8 nm. (d) Strain tensor map calculated from the second derivatives of the AFM. (e) Representative spectra of the LE and *X*_s_ maxima. The inset compares the LE emission from the nanopillar shown here and the one from Figure 3. (f) Topographic profile and *X*_s_ intensity plotted along the pink dotted line in (a) following the height gradient across the nanopillar. The intensity maximum occurs at the periphery of the height maximum, decaying less than 50% over 400 nm—indicating moderate localization. (g) Energy and intensity of the *X*_s_ plotted along the same line, showing that the emission maximum coincides with the energy minimum. (h) Comparison of topographic profiles between the nanopillars in Figures 3 and 4, illustrating different height variations. (i) Energy shift for a spatial variation 2.5 nm for the LE peak from the symmetric nanopillar and the *X*_s_ for the distorted nanopillar are plotted for the line plot displayed in Figure 3e and (g), distinguishing their strain gradient. All scale bars correspond to 200 nm.

Figure 4f,g show the spatial variation of both intensity and energy overlaid with the AFM topography. The maximum intensity and minimum energy coincide and occur outside the central top area of the pillar. However, both features show modest gradients: the energy varies by only 6 meV over 400 nm variation (compared to 17 meV in Figure 3 in 80 nm), and intensity remains above 50% throughout, indicating a weak confinement regime and resembling the confinement potential explanation which does not include the defect hybridization. However, the localized emission (LE = 1.53 eV, identical to that observed in the nanopillar of Figure 3) presents a very distinct confinement regime. It appears only in a sub-60 nm region with a sharply localized peak confined to a single pixel, rapidly vanishing outside that region. Figure 4e presents representative spectra from two distinct positions (pointed in Figure 4b) within the pillar, highlighting the dominant *X*_s_ feature and the coexistence of *X*_s_ and the LE. The inset compares the LE spectra from Figures 3 and 4, showing matching emission energies, despite the pronounced morphological differences between the two pillars.

The LE in Figure 4 also exhibits its intensity maximum near a local topographic peak, however in a specific region, as shown in Figure 4c and illustrated in the 3D AFM from SI Figure 5. Figure 4h compares the topographic profiles of the well-formed nanopillar (labeled as symmetric in purple) from Figure 3 with the distorted structure of Figure 4 (green). The height gradient in the former is significantly steeper, with a median slope of 2.01 nm of height variation per nanometer of lateral displacement, whereas the distorted pillar exhibits a much gentler slope of only 0.06. This difference is further confirmed in Figure 4i, where the energy shift over a 2.5 nm interval for the LE (symmetric pillar) and *X*_s_(distorted pillar) is plotted, revealing that the well-formed pillar induces more than four times the energy variation compared to the distorted one. Such disparity in strain gradients plays a crucial role in exciton transport: as neutral quasiparticles, excitons tend to diffuse more efficiently in the presence of sharper energy landscapes. Accordingly, the weak and anisotropic strain field in the distorted pillar is insufficient to generate strongly confined *X*_s_ emission centers, as predicted by theory.^30,31,34^ This will be further explored in the following case, which presents a less defective morphology. Nevertheless, the observation of a strongly localized LE emission in this structure—despite its shallow topography and spatial detachment from the *X*_s_ region—points again to an alternative localization mechanism. This behavior is most likely driven by hybridization with a local defect,^33^ in analogy with the scenario observed in Figure 3.

To further explore how anisotropic geometries affect strain propagation and emission profiles, a distinct distorted nanopillar was analyzed providing valuable insights into strain propagation and exciton diffusion. Figure 5a presents the energy shift of the *X* ^0^ exciton in a highly anisotropic structure, where the lateral dimension along the *x*-axis is significantly smaller than along the *y*-axis (*x* = 185 nm, *y* = 530 nm). Notably, the nanoPL map indicates that strain propagates more efficiently along the *x*-direction, where a blue shift remains observable up to 300 nm beyond the pillar boundary. In contrast, the *y*-direction shows a much shorter strain gradient, likely due to its larger physical dimension. Interestingly, the most pronounced energy shift for the *X* ^0^ occurs within the vertical edges of the nanopillar, suggesting that the narrower cross-section induces a stronger local strain field with a maximum at the borders rather than the apex, in good agreement with earlier reports.^30,31^ In line with previous observations, SI Figure 7a shows that the *X* ^0^ intensity is significantly reduced within the nanopillar, clearly delineating its spatial extent. However, this reduction does not imply antifunneling behavior. As shown in SI Figure 7e, the total quantum yield is in fact enhanced when the contribution from the *X*_s_ emission is taken into account.

**Figure 5:**
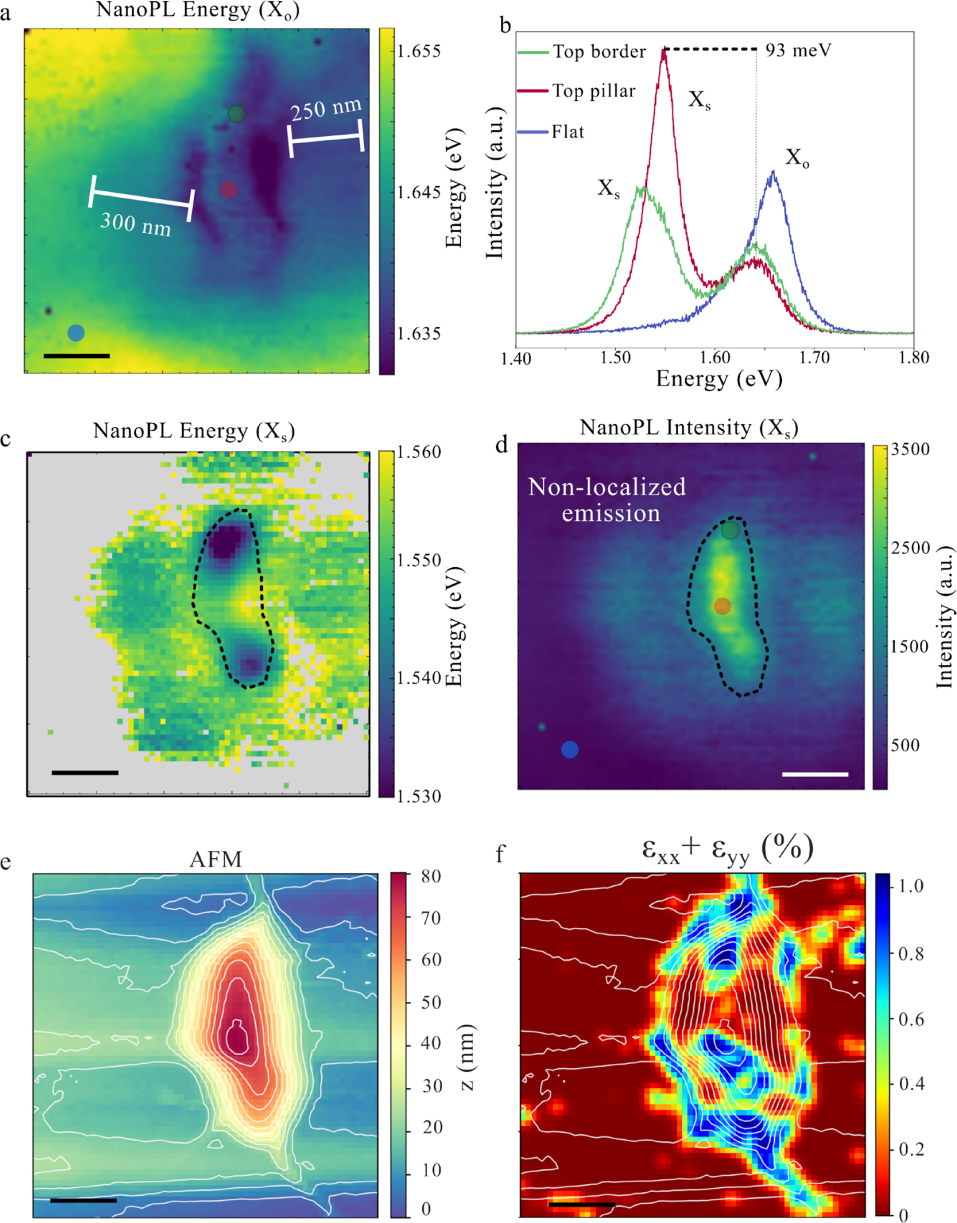
Distorted nanopillar 2 – single apex only (a) NanoPL energy map of *X* ^0^, showing strain propagation up to ∼300 nm beyond the nanopillar with preferential orientation along the shorter length axis of the nanopillar; the lowest-energy peak appears laterally. (b) Spectra from three distinct regions highlighted with faded dots with matching colors in (a, d), displaying both *X*_s_ and *X* ^0^ peaks, including their energy shifts and splitting. (c) NanoPL energy map of *X*_s_, with lowest-energy emission concentrated at the top and bottom corners, aligned with the pillar’s elongated axis. (d) NanoPL intensity map of *X*_s_, indicating diminished exciton localization due to a reduced strain gradient. (e) AFM topography of the nanopillar. The white contour lines represent height variations of 5.7 nm. (f) Strain tensor colormap (ϵ_*xx*_ + ϵ_*yy*_) for the second distorted nanopillar. All scale bars represent 200 nm.

Figure 5b shows spectra acquired from three representative positions indicated with faded circles in Figure 5a,d. The spectra reveal a 93 meV energy separation between the *X* ^0^ and *X*_s_ peaks, along with a pronounced broadening of the *X*_s_ line width at the main emission sites possibly attributed to the overlap of multiple lower-energy states. Notably, no LE emission is detected in this pillar. We emphasize that, while the *X*_s_ energy varies substantially among different nanopillar structures compatible with the different landscapes, the LE emission, when present, consistently appears at the same energy and remains strongly confined to small regions within the nanopillar. In contrast, the *X*_s_ emission often extends beyond the pillar boundaries. Figure 5c,d illustrate the behavior of the *X*_s_ emission, which exhibits two distinctive features. First, the lowest-energy emission appears along the upper and lower edges of the structure, whereas the emission exhibits a blueshift at the vertical borders. These opposite energy responses indicate that *X*_s_ and *X* ^0^ respond differently to the local strain field, with the possibility of responding differently to compressive and tensile strain. Second, the region of highest *X*_s_ intensity does not coincide with its lowest energy, unlike in the previously investigated nanopillars. Instead, the intensity maximum is distributed along the nanopillar (see SI Figure 8 for line plots) and extends over a relatively large area. This behavior can be understood by considering the AFM topography and the calculated strain trace shown in Figure 5e,f. Although the overall slope of the topography is similar to that of the nanopillar shown in Figure 4 with a moderate height gradient of approximately 0.05 (corresponding to a height variation of about 0.05 nm per 1 nm in the lateral direction), the present structure features a single, well-defined apex as seen in the white contour lines (Figure 5e,f), whereas the previous example contained at least three local maxima (see Figure 4c for comparison). As a result, the calculated strain map reveals larger strain values than in the previous nanopillar, consistent with the lower energy of the *X*_s_ emission, and a broader spatial distribution of strain. Moreover, finite strain is also predicted in regions close to the nanopillar top and center, in contrast to the other strain maps that display extended near-zero areas at the apex. This difference likely reflects enhanced local strain inhomogeneities also suggested by the higher contour-line density in the AFM topography. While this strain distribution is compatible with an extended spatial character of the *X*_s_, the calculation is insufficient to account for the distinct spatial locations of the *X* ^0^ and *X*_s_ energy shifts. This discrepancy is further compounded by the limitations of the present model, which does not reliably capture compressive and shear strain components that are expected to play a role in such distorted nanopillar geometries. Despite these limitations, it is important to emphasize that, although the distorted nanopillar shown in Figure 5 exhibits comparable or even higher strain—as inferred from similar *X* ^0^ energy shifts of 25–30 meV when compared to Figure 2c—no LE emission is observed. This observation further reiterates that strain alone is not sufficient to induce LE states, highlighting the necessity of additional conditions for their formation.

Interestingly, for the well-rounded nanopillar shown in Figure 2, no lower-energy emission is detected outside the nanopillar region. This suggests that, in highly symmetric configurations, strain relaxation and TMD conformation are more uniform, favoring the confinement of lower-energy emission to the nanopillar periphery and to wrinkle-like regions. It is important to emphasize that the strain model employed here, based on curvature analysis as described in ref 48, is primarily sensitive to out-of-plane deformation; consequently, strain components that do not produce measurable curvature, such as residual in-plane or lattice-scale distortions in flat regions, are not captured by the calculation. In this sense, NanoPL provides a more reliable probe of local strain effects than the simplified continuum model used in this work. Although NanoPL measurements at cryogenic temperatures are not accessible in our setup and the temperature evolution of the spatial emission distribution cannot be directly assessed, these observations nonetheless highlight the crucial role of nanopillar geometry in exciton confinement and the potential formation of SPE states within the pillar. In contrast, in less symmetric structures, strain-induced localized excitonic states may extend beyond the nanopillar boundaries, as observed in Figure 5c. Overall, our results demonstrate how nanopillar design directly influences the optical response, offering practical guidance for optimizing strain-engineered nanopillar architectures.

### Platform

An important insight emerging from the previous analyses is the dominant role of strain in activating the *X*_s_ emission, as opposed to being a residual artifact of the nanopillar fabrication process or variations in material composition. To further investigate this effect in a different structural context, we designed a step-like control sample consisting of a micrometer-scale platform fabricated using the same lithographic process as the nanopillars. This structure maintains a similar height profile (160 nm) but extends laterally over a much larger area, removing the influence of nanoscale confinement inherent to dot-like nanopillars. Figure 6a shows the AFM topography of the platform, revealing the central elevated region along with three wrinkle-like features generated during the lithography exposure. The corresponding NanoPL intensity map at the LE energy (Figure 6b) demonstrates that low-energy emission arises exclusively at the platform edges and along the wrinkles, regions where localized strain fields are expected to concentrate. In contrast, the central, unstrained flat area exhibits no detectable lower energy emissions. This spatial emission pattern further supports the conclusion that local strain is the primary mechanism responsible for LE activation in these structures. While NanoPL clearly resolves these emissions both spectrally and spatially, the far-field measurement (SI Figure 9) fails to resolve the low-energy feature in the spectrum and provides no information on its spatial localization. Similar to the other analyzed structures, the strain tensor was calculated and its trace is shown in Figure 6c, closely reflecting the AFM topography. In this case, the platform exhibits a more conformal and quasi–one-dimensional topography, in contrast to the complex and highly distorted nanopillar geometries. For such simplified and symmetric configurations, the strain model yields physically consistent and reliable results, as the strain distribution is predominantly governed by well-defined curvature, enabling a more direct correspondence between topography and strain. Notably, the wrinkles, due to their narrow lateral dimensions, induce more localized strain fields and consequently support more confined LE emission with higher intensities—comparable to those observed at the platform edge, even though they present lower height variation (wrinkles with 30 to 50 nm height and the platform ≈100 nm) as also reflected in the strain map. This contrast is evident in the NanoPL color map (Figure 6b) and corresponding spectra for four representative regions displayed in Figure 6d, where emission at the wrinkle sites is sharper and more spatially confined than at the broader interface region, while no lower-energy emission is detected in the flat regions.

**Figure 6:**
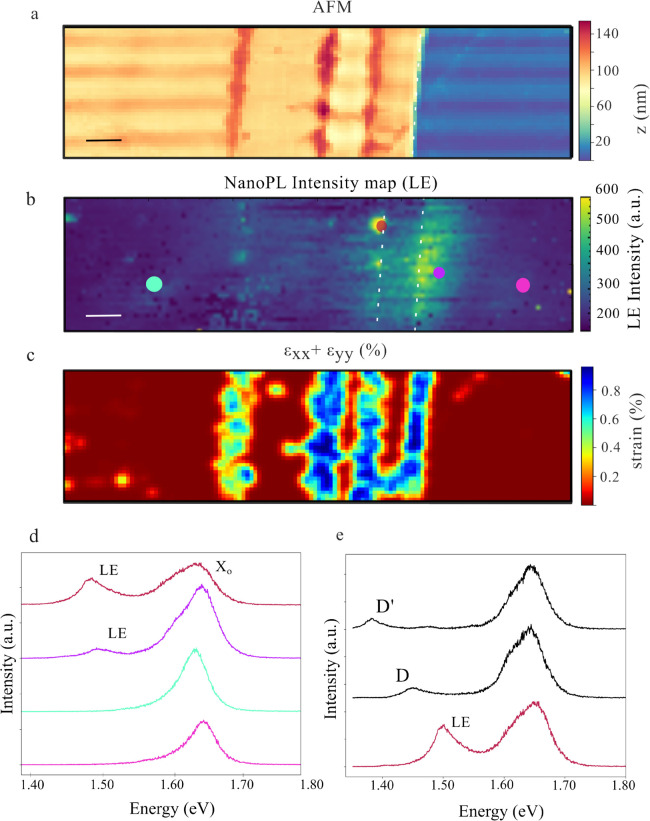
Step-like platform. (a) AFM topography of the platform showing an abrupt height variation of ≈100 nm and three fine wrinkles, with heights ranging from 30 to 50 nm. (b) NanoPL intensity map of the LE, spatially confined to the strained regions created by the wrinkles and at the platform edge. (c) Trace tensor map derived from the AFM. (d) NanoPL spectra from selected locations, with colors matching the markers in (b). (e) NanoPL spectra acquired, revealing two lower-energy features (D and D’) restricted to a single pixel, attributed to defect-related emission. All scale bars correspond to 200 nm. Colormaps for the defect emission is depicted in the SI Figure 10.

In addition to the strain-activated LE, faint sub-bandgap features are detected at isolated positions within the platform. These emissions are confined to single-pixel resolution and exhibit very low intensities, as shown in the spectra of Figure 6e, labeled D and D’ (see SI Figure 10 for the colormap for D and D’). Their highly localized nature, weak dipole strength and lack of correlation with the calculated strain distribution strongly suggest an origin associated with defect states. This observation further demonstrates the sensitivity of the NanoPL setup to probe nanoscale inhomogeneities. Moreover, these features highlight the potential to investigate hybridization between defect states and dark excitonic transitions in strained 2D materials.

## Conclusions

In this work, we directly visualized the photoluminescence response of strain-engineered WSe_2_ nanopillar structures using tip-enhanced photoluminescence, achieving high-resolution spectra that uncover the confinement mechanisms underlying possible single-photon emitter formation. Our spatially and spectrally resolved NanoPL maps associated with tensor strain calculations via AFM reveal how local strain distribution, topographic features, and nanoscale defects act together to govern exciton localization and diffusion. We identified two distinct confinement regimes: (i) potential-well confinement driven by nonuniform nanoscale strain distributions beyond the reach of continuum strain models, leading to efficient exciton diffusion and strong *X* ^0^ quenching at the nanopillar periphery; and (ii) defect-mediated confinement activated by strain, characterized by energetically well-defined low-energy states with strong spatial localization down to ∼10 nm, exhibiting pronounced sensitivity to local strain variations and energy shifts consistent with hybridization with dark excitonic states, as commonly associated with SPEs at cryogenic temperatures. We demonstrate that symmetric nanopillars with an aspect ratio of ≈1.17 support stronger confinement, while lower-aspect-ratio, anisotropic structures require combined complex topography with multiple apexes for efficient localization. From our data, we extract a characteristic energy variation regime of 0.5–1.0 meV/nm as a practical metric for LE (and likely SPE-related) emission, in contrast to a significantly smaller energy variation of approximately 0.25 meV/nm for strain pocket theory. Moreover, distorted nanopillars enabled quantification of exciton diffusion lengths up to 300 nm beyond the structure, offering deeper insight into the interplay between geometry and emission. These findings provide, to our knowledge, the most detailed spatial characterization to date of localized excitonic states confined within nanopillar boundaries. Based on these observations, our nanoPL maps provide a robust and direct probe of the local optical response in different strained nanostructures. For complex and highly asymmetric geometries, where strain modeling remains incomplete, nanoPL currently represents the most reliable approach to describe the underlying excitonic landscape. Overall, our study not only validates theoretical predictions but also defines key structural parameters for designing strain based devices.

## Experimental Section

### Sample Information

Nanopillar arrays were fabricated on *SiO*_2_ substrates by electron-beam lithography using a PMMA (poly­(methyl methacrylate)) resist. To tune the nanopillar height, different PMMA formulations (C2 and C4) were employed, yielding polymer films with thicknesses in the range of 100–200 nm, spin-coated at a fixed rotation speed of 4000 rpm with an acceleration of 1000 rpm/s. High exposure doses (3–120 pC) were applied in dot-writing mode to induce polymer cross-linking, resulting in transparent nanostructures with heights between approximately 100 and 160 nm, depending on the initial film thickness. These structures remained firmly anchored to the substrate and were resistant to conventional solvents (see inset of Figure 1a). To achieve smoother and more symmetric pillar tops, the electron-beam focus and astigmatism were carefully optimized, using neighboring pillars as alignment references. The lateral dimensions of the nanopillars were controlled by the exposure dose: for example, a dose of 100 pC produced pillars with diameters of approximately 338 nm, whereas a 10 pC dose yielded structures around 260 nm in diameter. Overall, the nanopillars investigated in this work exhibited diameters ranging from 160 to 300 nm.

Commercial WSe_2_ flakes were mechanically exfoliated using commercial blue tape and subsequently transferred via standard dry stamp-and-stack transfer method using polycarbonate adhere to PDMS^53^ and temperature control. For the samples studied, a temperature of 70 °C were use to pickup hBN and WSe_2_ and a temperature of 170 °C were use to deposit the heterostructure on top of the nanopillars. The nanopillars were also transferred from *SiO*_2_ to quartz using the same technique but applying lateral force during the transfer process through the PDMS stamp. Nanopillars were also directly produced in the quartz substrate, which resulted in malformed nanopillars studied in the manuscript.

### Optical Measurements

Tip-enhanced photoluminescence measurements were carried out using the custom-built Porto laboratory setup, equipped with an oil-immersion objective lens (NA = 1.4) and a radially polarized He–Ne laser (632.8 nm) operating at 1.9 μW. The metallic tips employed in the experiments were Plasmon-Tunable Tip Pyramids.^27^ Spectral data analysis and hyperspectral image processing were performed using the PortoFlow Analysis Software (version 1.19). Atomic force microscopy images were acquired simultaneously with the NanoPL measurements using a tuning-fork shear-force feedback, ensuring precise spatial correlation between optical and topographical data. Under our experimental conditions, Purcell enhancement effects are expected to be negligible, since the TEPL tip is plasmonically resonant with the excitation wavelength rather than the emission energies, the setup does not operate in a gap-mode geometry or incorporate metallic substrates, and the strong spectral detuning of the low-energy emission further suppresses any cavity-induced modification of the local photonic density of states. Additionally, The tip–sample distance is controlled by shear-force AFM, where the picoNewton-scale interaction forces are several orders of magnitude smaller than the nanoNewton-scale forces responsible for strain formation in the WSe_2_ monolayer, ensuring negligible mechanical perturbation of the sample.

Low-temperature (4 K) photoluminescence measurements were conducted in a confocal microscope coupled to a AttoDry magneto-cryostat. Excitation was achieved using 660 and 730 nm lasers, the latter being close to the defect-resonant excitation wavelength. The excitation beam was focused onto the sample through an objective lens with a numerical aperture of 0.68, and the backscattered signal was collected and directed to a spectrometer equipped with a high-sensitivity CCD detector for PL acquisition. To optimize the visualization of excitonic doublets, different diffraction gratings were employed depending on the required spectral resolution: 150, 600, and 1200 lines/mm.

### Data Processing

All data processing, including the spectral fitting of color maps, was performed using the Fabns PortoFlow software. Photoluminescence peaks were fitted with Gaussian profiles through a least-squares optimization method.

## Supplementary Material



## Data Availability

The raw data underlying the analyses in this manuscript are available at https://doi.org/10.7910/DVN/BXHLFD Harvard Dataverse data repository.

## Notes

Strain calculation method; comparison between far-field and near-field measurements; additional analyses of the experimental data; and defect colormaps (PDF)
