# Supplementary material for: Quantum Confinement Emissions in Strained Monolayer WSe2: A Nanoscale Approach to Single-Photon Emitters via Tip-Enhanced Techniques
Source: ACS Nano. 2026 Mar 21;20(13):10381–93. doi: 10.1021/acsnano.5c18642 (PMC13063805; doi:10.1021/acsnano.5c18642)
Supplement: Supplementary file 1 [file nn5c18642_si_001.pdf]

Supplementary Information: Quantum Confinement Emissions in Strained Monolayer WSe_2: A Nanoscale Approach to Single-Photon Emitters via Tip-Enhanced Techniques

Lucas Liberal[1,2], Rafael Battistella Nadas[1,3], Gustavo H. R. Soares[2,4], Frederico B. Sousa[2], Maria Clara Godinho[1], Gabriel Marques Jacobsen[2], Takashi Taniguchi[5], Kenji Watanabe[6], Marcio Daldin Teodoro[2], Ado Jorio[1], Leonardo Cristiano Campos[1,7]

[1]Departamento de Física, Universidade Federal de Minas Gerais, Belo Horizonte, MG, 31270-901, Brazil

[2]Departamento de Física, Universidade Federal de São Carlos, São Carlos, SP, 13565-905, Brazil

[3]Institut für Physik, Humboldt-Universität zu Berlin, Newtonstraße 15, Berlin, 12489, Germany

[4] Institut für Experimentelle und Angewandte Physik, Universität Regensburg, Regensburg, 93053, Germany

[5]Research Center for Materials Nanoarchitectonics, NIMS, Tsukuba, 305-0044, Japan

[6]Research Center for Electronic and Optical Materials, NIMS, Tsukuba, 305-0044, Japan

[7] Centro de Tecnologia em Nanomateriais e Grafeno, Belo Horizonte, MG, 31310-260, Brazil

*

E-mail: lucas.liberal.fonseca@gmail.com

Strain calculation

The strain profile associated with an extended membrane deformation is obtained by modeling the monolayer as a thin elastic plate whose out-of-plane displacement is directly measured by atomic force microscopy (AFM). In this framework, the experimentally measured height profile $h(x, y)$ is treated as a known input, while the in-plane elastic response of the membrane is inferred from continuum elasticity theory.

Mechanical equilibrium for an elastic continuum requires that internal stresses balance the forces applied at the boundaries. In the absence of body forces, this condition can be expressed as

$$\sum_k \sigma_{ik} n_k = P_i, \tag{1}$$

where P_i denotes the applied traction (pressure in the present case), σ_{ik} are the stress tensor components, and n_k is the unit normal to the surface.

For an atomically thin membrane, only small forces are required to induce substantial out-of-plane bending relative to the in-plane elastic stresses. As a result, stress components involving the surface-normal direction can be neglected. Assuming that the surface normal lies along z, this leads to the plane-stress condition

$$\sigma_{zz} = \sigma_{xz} = \sigma_{yz} = 0,$$

such that the elastic response is fully described by the in-plane stress components.

Under plane-stress conditions, the constitutive relations follow Hooke's law for an isotropic elastic plate,

$$\begin{aligned} \varepsilon_{xx} &= \frac{\sigma_{xx} - \nu\sigma_{yy}}{E}, \\ \varepsilon_{yy} &= \frac{\sigma_{yy} - \nu\sigma_{xx}}{E}, \\ \varepsilon_{xy} &= \frac{(1+\nu)\sigma_{xy}}{E}, \end{aligned} \tag{2}$$

where ε_{ij} are the components of the strain tensor, E is the Young's modulus, and ν is the

Poisson ratio.

The strain tensor is defined in terms of the displacement from a reference configuration. The total displacement is decomposed into an in-plane vector $\mathbf{u}(x, y)$ and an out-of-plane displacement $h(x, y)$. To account for geometric nonlinearities arising from bending while assuming small in-plane strains, the Green–Lagrange formalism is adopted. In this description, the strain components include linear terms involving gradients of $\mathbf{u}$ and quadratic terms involving gradients of $h(x, y)$.

Mechanical equilibrium additionally requires that the divergence of the stress tensor vanishes in the absence of body forces,

$$\nabla \cdot \boldsymbol{\sigma} = 0.$$

This condition is satisfied by introducing a scalar Airy stress function $\chi(x, y)$, such that

$$\sigma_{xx} = \frac{\partial^2 \chi}{\partial y^2}, \quad \sigma_{yy} = \frac{\partial^2 \chi}{\partial x^2}, \quad \sigma_{xy} = -\frac{\partial^2 \chi}{\partial x \partial y}. \tag{3}$$

Combining the constitutive relations, the strain–displacement relations, and the Airy stress formulation reduces the problem to a fourth-order partial differential equation for χ:

$$\nabla^4 \chi = -E \left(\frac{\partial^2 h}{\partial x^2} \frac{\partial^2 h}{\partial y^2} - \left(\frac{\partial^2 h}{\partial x \partial y} \right)^2 \right), \tag{4}$$

where the source term corresponds to the Gaussian curvature of the measured height profile.

To solve this equation numerically, a spectral collocation method based on Chebyshev polynomials is employed. The AFM topography is first fitted with a smooth function to ensure numerical stability in evaluating higher-order derivatives. The Gaussian curvature is then computed at the Chebyshev collocation points, which define the source term of the governing equation. Differential operators are represented as matrices constructed from Chebyshev derivative matrices, mapping the continuous problem onto a linear algebraic

system.

Physically motivated boundary conditions are imposed by assuming that, far from the deformation, both $h(x, y)$ and its gradient vanish. This corresponds to a traction-free boundary without externally applied in-plane stress. Under these conditions, the Laplacian of χ is set to zero at the boundary. Additional constraints are applied directly to χ to eliminate non-physical solutions and stabilize the numerical procedure.

Once the Airy stress function is obtained at the collocation points, it is interpolated back onto a real-space grid matching the AFM data. The spatially resolved strain tensor components are then computed from second derivatives of χ according to the plane-stress relations, yielding quantitative maps of the in-plane strain field associated with the measured topography.

The overall approach treats the AFM profile $h(x, y)$ as a prescribed geometric input, modeling the monolayer as an isotropic elastic membrane under plane-stress conditions and without resolving atomistic degrees of freedom as in a tight-binding framework. The accuracy of the reconstructed strain tensor depends sensitively on the smoothness of $h(x, y)$ and on the imposed boundary conditions. Notably, the shear strain component ε_{xy}, which depends on mixed second derivatives, is particularly susceptible to experimental noise and often displays rapid sign variations, limiting its interpretability compared to the remaining strain components. Consequently, the method provides reliable access to the principal components of the in-plane strain for smoothly varying deformation profiles, while its descriptive power may diminish for topographies featuring elastic anisotropy or deviations from linear continuum elasticity.

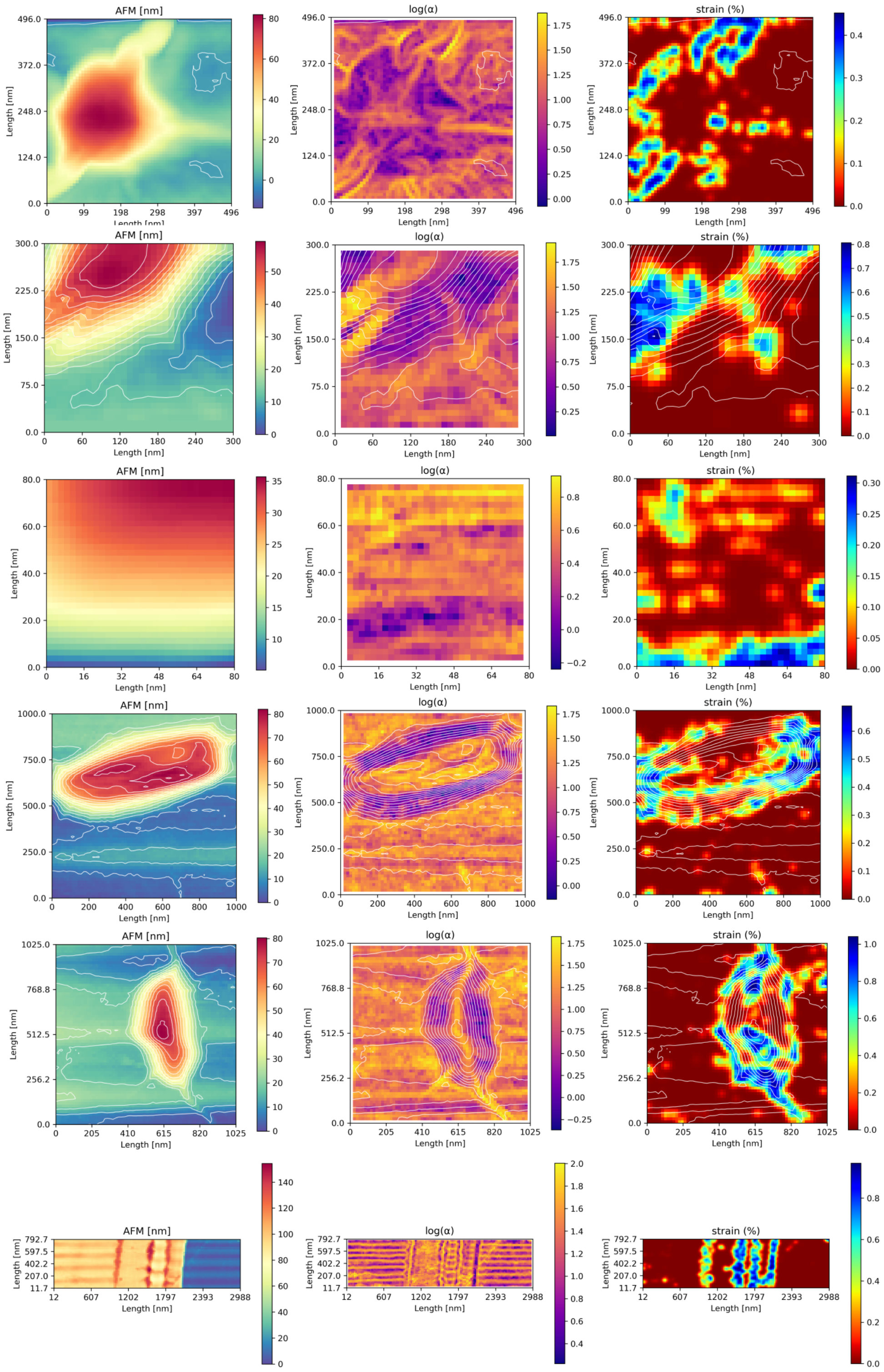

SI Figure 1 — Strain calculation. AFM topography, curvature map expressed as log(α), and corresponding strain colormaps for all studied nanopillar geometries. The parameter α is obtained from the AFM topography and is proportional to the local curvature of the membrane, being derived from second-order spatial derivatives of the height profile. The logarithmic scale is used to enhance contrast and allow the visualization of regions with both weak and strong curvature within the same map. The first nanopillar corresponds to the symmetric geometry analyzed in Figs. 2 and 3, which closely resembles the nanopillars investigated under low-temperature conditions.

NanoPL and Far field Subtraction

All NanoPL colormaps presented in this manuscript represent the result of subtracting the far-field (FF) spectrum from the near-field (NF) spectrum, thereby isolating the enhancement effect induced by the tip and obtaining the corresponding local behavior. This can be done because in the backscattered measurement configuration, the tip-down measurement is a sum of NF and FF signals. Thus, right after the tip-down map is done, we retract the tip (tip-up, around 1 um away) and repeat the experiment with the same experimental parameters (number of pixels, acquisition time per pixel and physical size). Therefore, to isolate the NF component of the signal, we subtract the tip-up from the tip-down signal. This is fundamental for photoluminescence studies, since the enhancement is small when compared to Raman spectroscopy.

Far Field and Near Field comparison of the map of Fig. 2

Far-field measurements cannot spatially resolve the emission within the nanopillar due to their limited spatial resolution. As a result, none of the effects discussed in Fig. 2 can be directly observed in the far field, thereby reinforcing the necessity of tip-enhanced measurements.

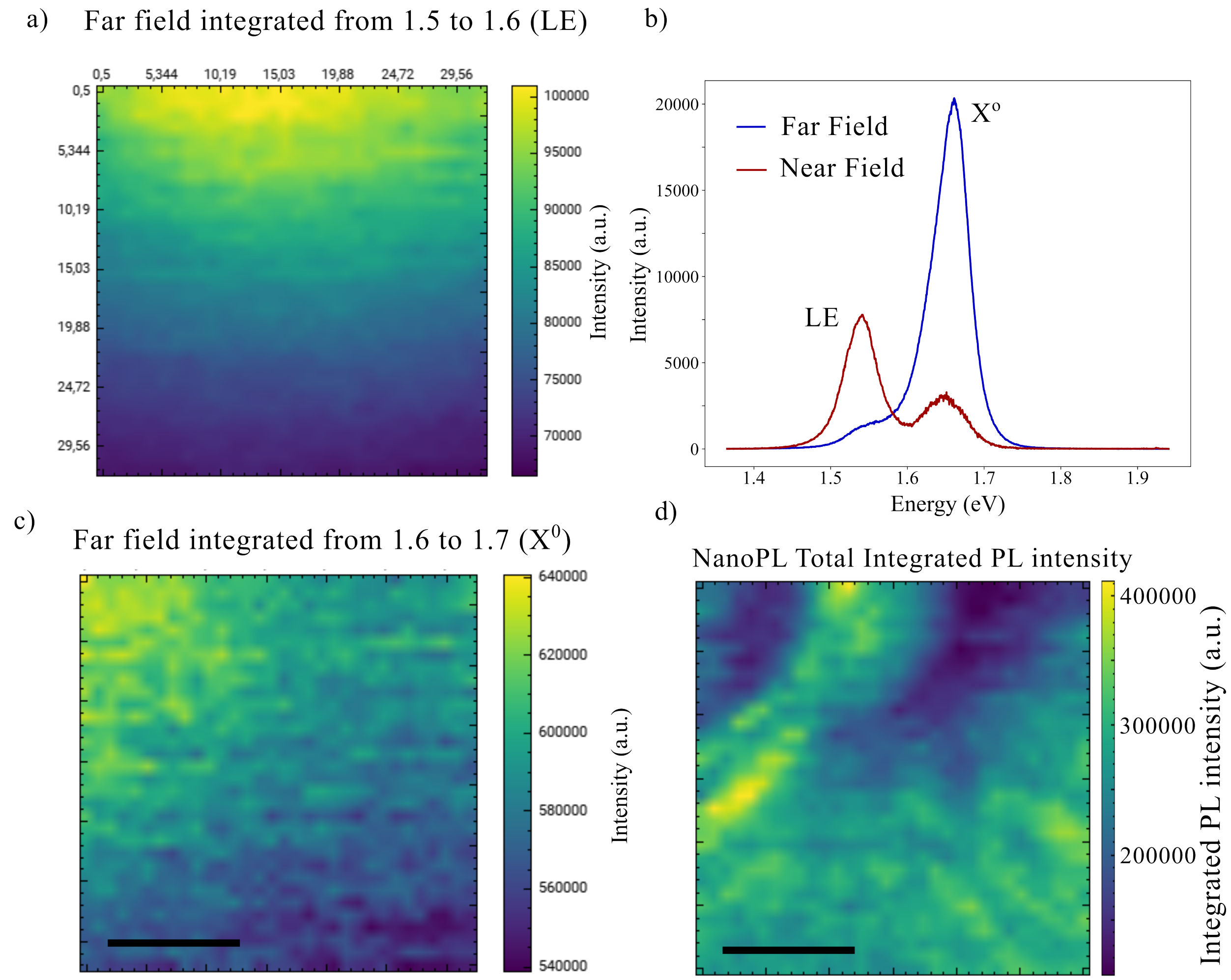

SI Figure 2 | a) Far-field colormap for the LE and b) a representative spectrum corresponding to Fig. 2. The near-field signal shown is obtained by subtracting the far-field contribution from the total measured signal. c) Far field colormap intensity for the X^0 d) Total integrated NanoPL showing regions of higher quantum yield as wrinkles and the nanopillar.

Far Field and Near Field comparison for the colormap of Fig. 3

Far Field and Near Field comparison for the colormap of Fig. 3 and the almost homogeneous signal of X^0. Further interpratation of the X^o was compromised due to the NF - FF subtraction due to low intensity of X_o.

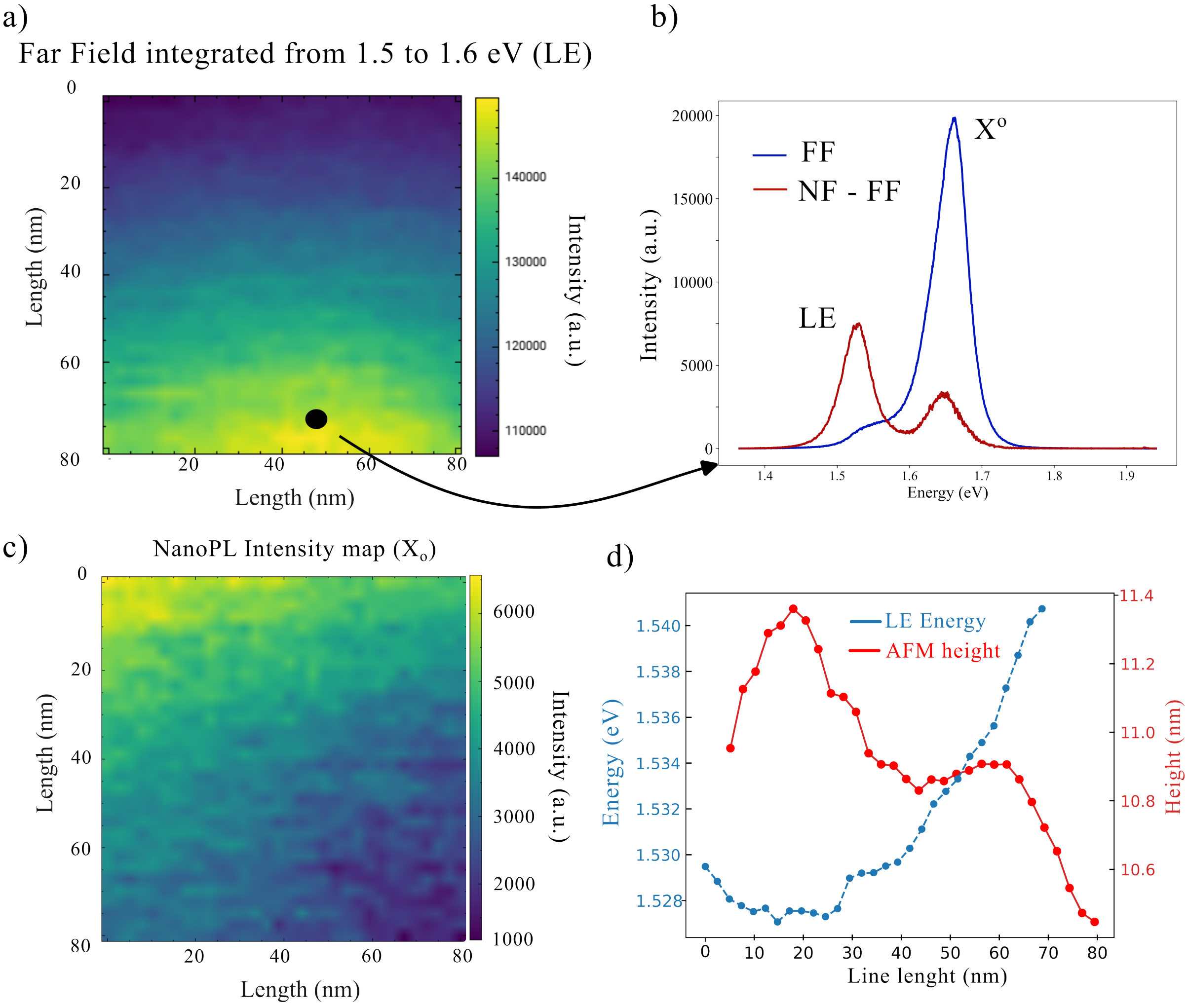

SI Figure 3 |(a) Far-field PL intensity integrated over the low-energy (LE) emission range. (b) Comparison between far-field and near-field (NF–FF) spectra, showing a marked difference: although far-field measurements collect LE emission over a larger area, they lack the spectral and spatial resolution required to accurately resolve its energy. (c) Spatial map of the X^o intensity, exhibiting enhanced emission near the nanopillar apex. (d) Horizontal line profile illustrating the dependence of the LE emission energy on the local height variation. An energy shift of approximately 12 meV is observed for a height variation of less than 1 nm.

Collapse of the symmetric nanopillar

Nanopillar burned not showing any PL and Raman signal after the increase in intensity of 19 μW in the tip enhancement regime. This limited a Raman investigation since Raman signal is weaker than PL in our sample so it was not possible to properly investigate it since

required a higher laser power than 1.9 μW. Due to this event, no Raman spectroscopy were performed in the subsequent samples.

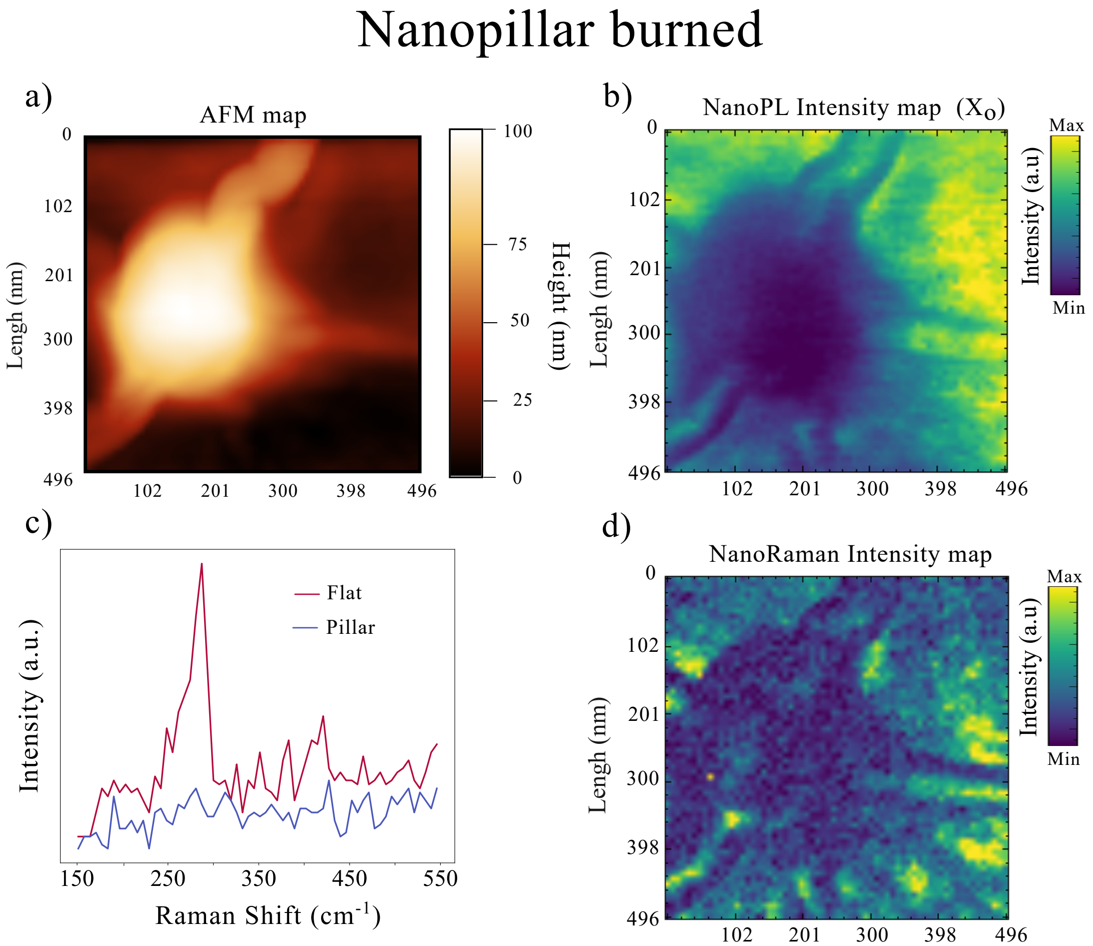

SI Figure 4 | (a) AFM topography. (b) Nano-PL map of the X_0 emission. (c) Raman spectrum. (d) Raman intensity colormap of the burned nanopillar. The measurements correspond to the same nanopillar analyzed in Figs. 2 and 3.

Supplementary discussion for the pillar in Fig. 4.

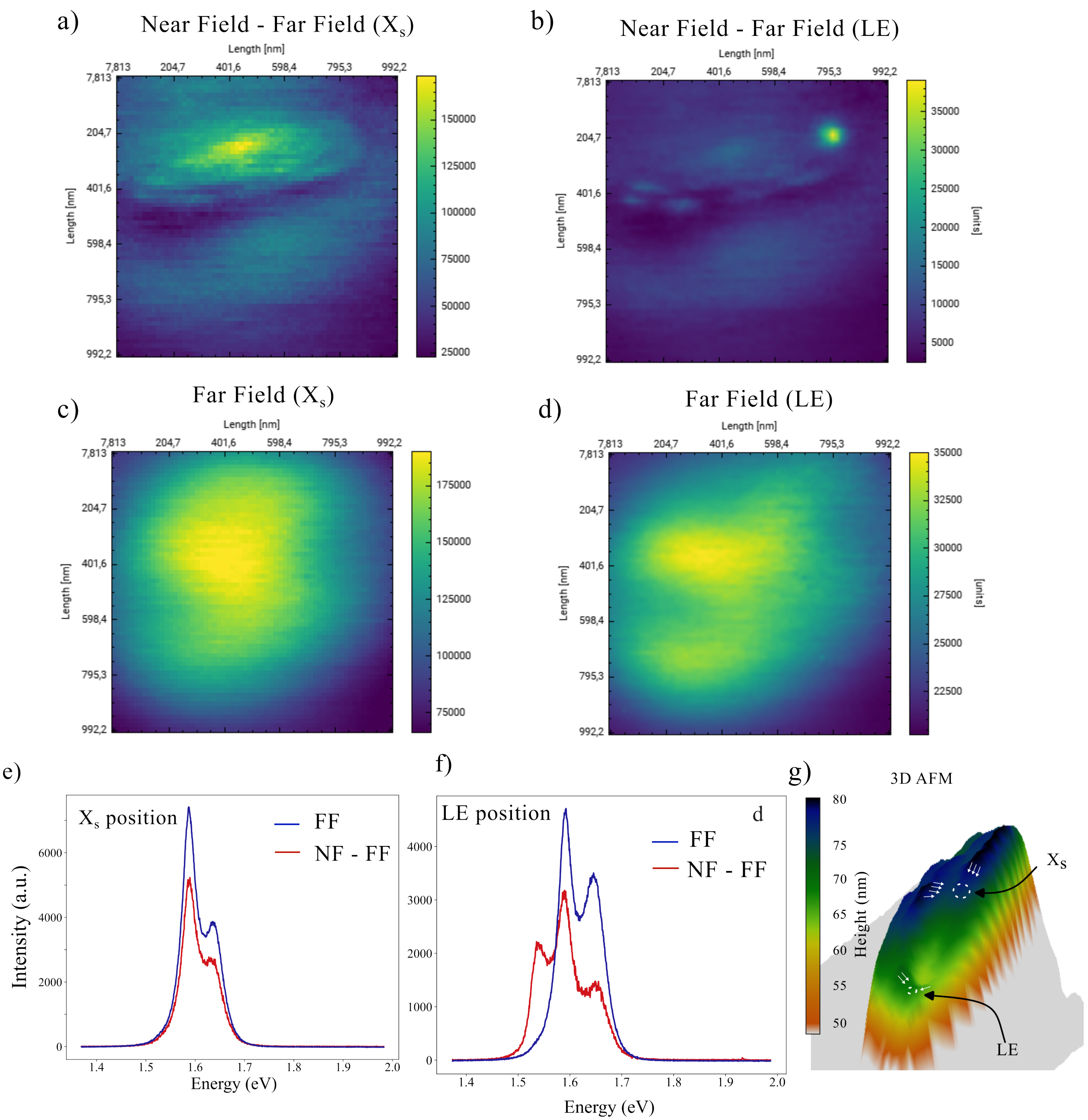

SI Figure 5 | Near-field (NF) intensity colormaps of the X_s (a) and LE (b) emissions. Far-field (FF) intensity colormaps of the X_s (c) and LE (d) emissions. Corresponding spectra of the X_s and LE emissions acquired at the positions of maximum intensity are shown in (e) and (f), respectively. (g) Three-dimensional AFM topography of the distorted nanopillar, highlighting the apex and the regions of maximum emission intensity.

As discussed in the main text, the X_s emission remains visible in the far-field spectrum because its intensity is distributed over a relatively large area. This behavior is in strong contrast to the localized emitter (LE), whose emission cannot be resolved in far-field spectra, as shown in panel (f). Although the far-field intensity map reveals the presence of the X_s emission, it is poorly spatially resolved and extends over an area significantly larger than the nanopillar itself, markedly differing from the near-field map. These observations further reinforce the necessity of nano-photoluminescence (NanoPL) measurements to properly resolve the spatial and spectral characteristics of localized emitters.

Consistent with other measurements, the X^0 emission is strongly suppressed within the nanopillar region, clearly delineating its spatial boundaries as seen in the figure below. When analyzing its energy profile, the neutral exciton is sensitive to topographical variations, exhibiting a minimum energy at the same location where the X_s reaches its maximum, precisely where an approximately orthogonal strain gradient is present. However, the emission intensity of the X^0 within the pillar does not reflect this energy modulation. This is likely due to competition with the X_s emission and the limited exciton diffusion, a consequence of the larger spatial extent of the nanopillar structure.

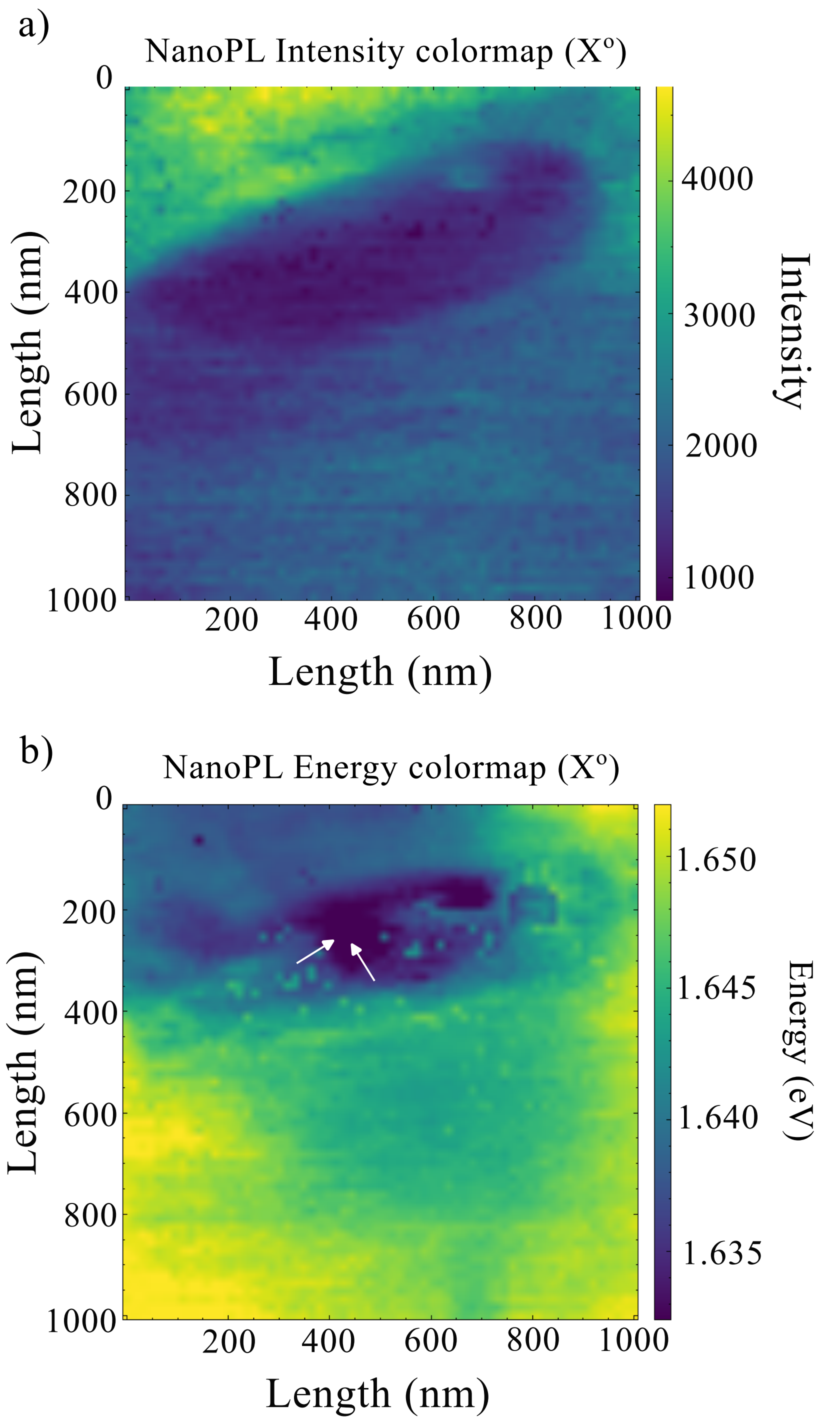

SI Figure 6| a) NanoPL intensity map for the X^0 b) NanoPL Energy map for the X^0.

Supplementary discussion for the pillar in Fig. 5

The NanoPL intensity map integrated over the entire spectral range captures the combined emission from both the neutral exciton (X^0) and the localized exciton (X_s). While the X^0 emission is strongest outside the nanopillar region, the X_s emission reaches its maximum intensity at the apex of the pillar—contrary to the behavior observed in other nanopillars. Near the edges of the pillar, both excitonic emissions show low intensity, despite the X^0 exhibiting its minimum energy there, as shown in Fig. 5. This behavior may be attributed to the anisotropic shape of the nanopillar, which presents a shorter x-dimension, similar to the structure in Fig. 2, known to promote efficient exciton funneling. However, due to its larger y-dimension, the X_s emission appears to spread preferentially along the y-axis, covering the full extent of the pillar, while presenting a more efficient exciton diffusion in the x-axis. In the far-field measurements, the X_s emission even seems to extend beyond the physical boundaries of the pillar, likely due to limited spatial resolution.

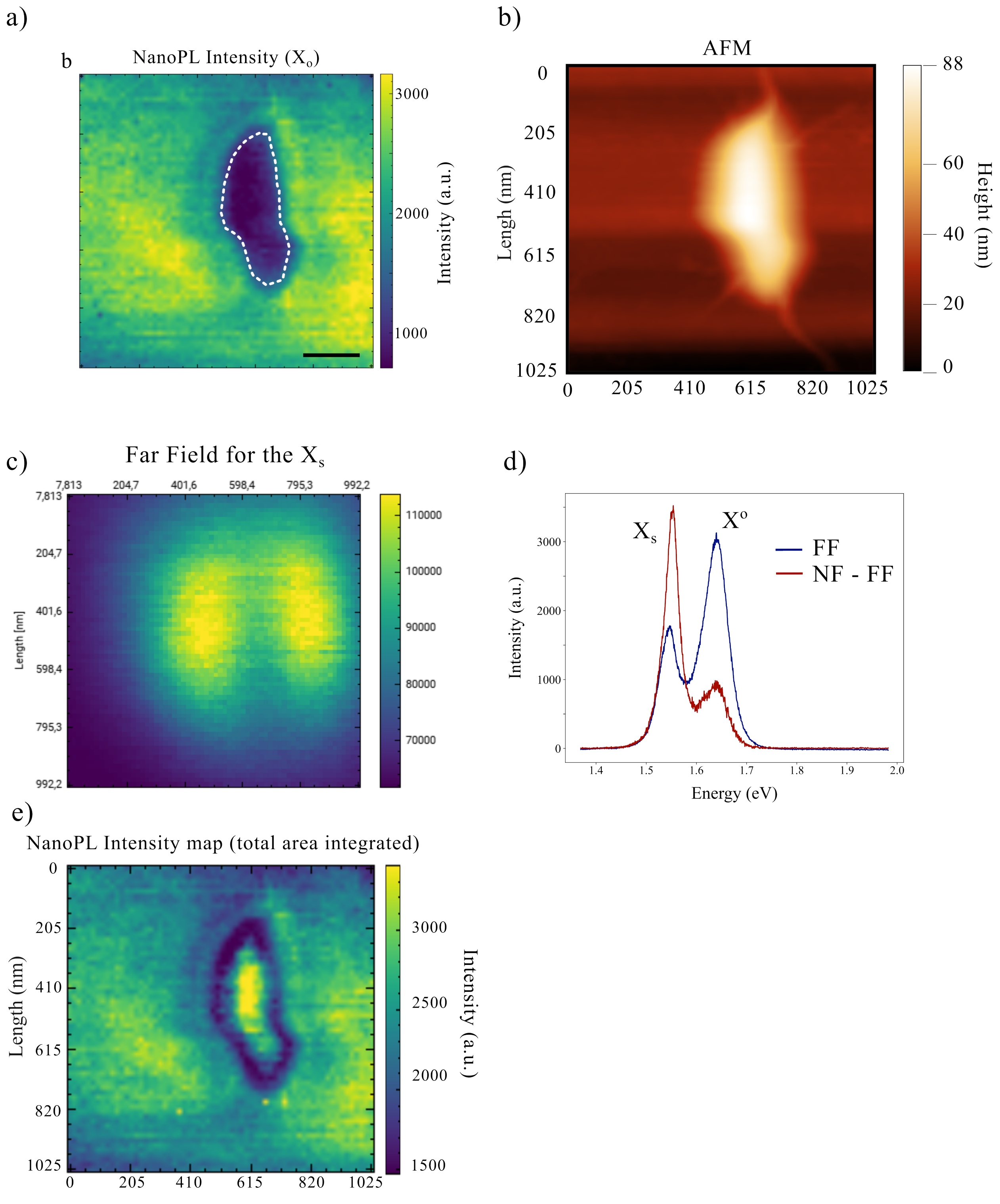

SI Figure 7| Additional information on the pillar from Fig. 5 (a) NanoPL intensity map for the (X^0) showing high quenching in the nanopillar region, clearly delimiting its extension (b) AFM measurement acquired simultaneously with the NanoPL map. (c) Far-field intensity colormap of the X_s, showing lower spatial resolution compared to the NanoPL map in Fig. 5. (d) Representative spectra comparing the far-field signal with the near-field–far-field subtraction. (e)NanoPL intensity colormap of the integrated area, showing enhanced quantum yield at the nanopillar center and reduced emission near its borders.

Line profiles of the X_s emission for pillar of Fig 5 reveal a distinct behavior compared to other nanopillars. In this case, the maximum intensity coincides with the region of highest X_s emission, which can be attributed to a gentler strain gradient that facilitates more efficient exciton diffusion. Notably, the emission peak spans a relatively large spatial extent, maintaining high intensity across a broad region.

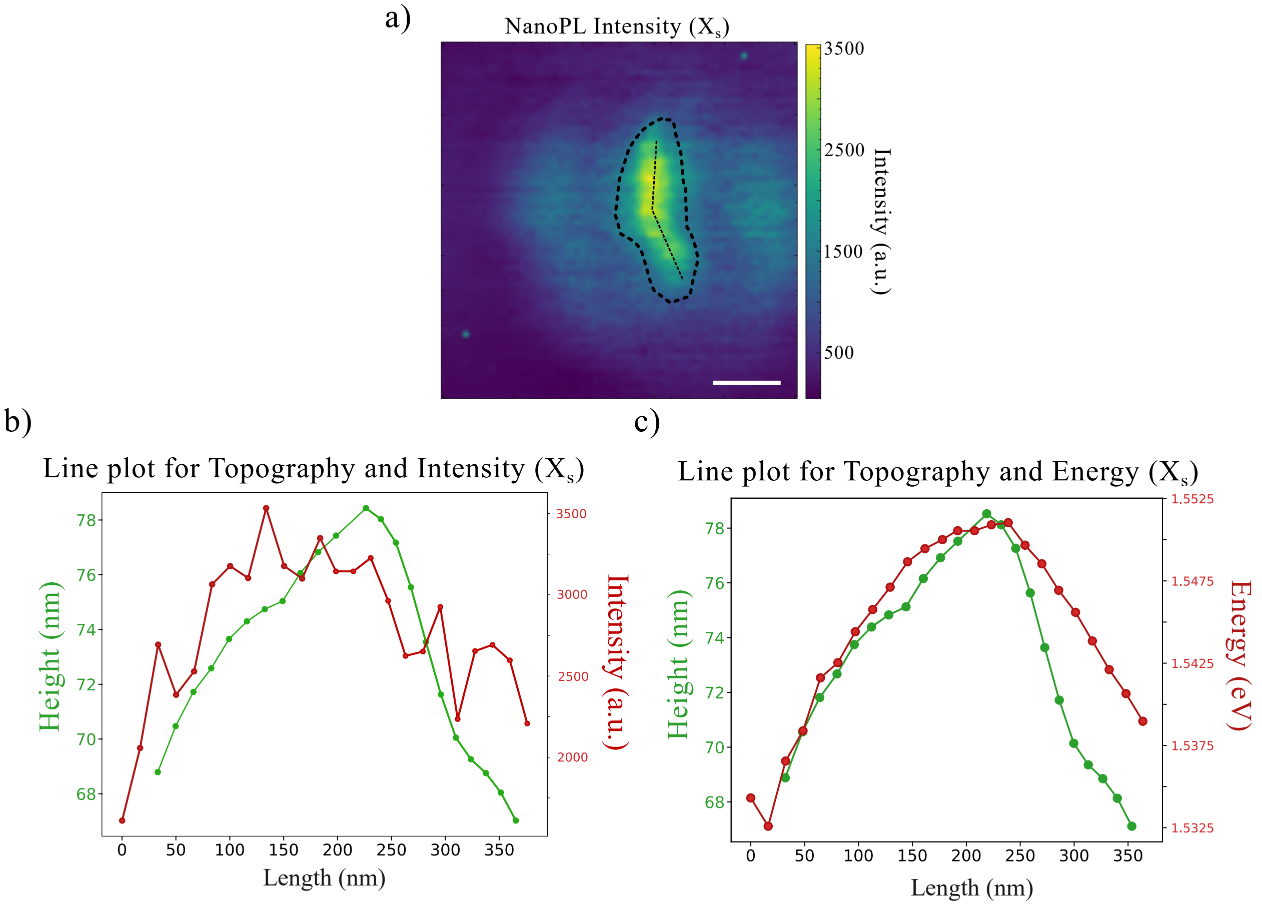

SI Figure 8| Line plot for pillar in Fig. 5 (a) NanoPL intensity colormap for X_s, corresponding to the data shown in Fig. 5 of the main text. (c),(d) Line profiles of intensity and energy as a function of line length, extracted along the dotted line indicated in (a).

Supplementary discussion for the step-like platform in Fig. 6

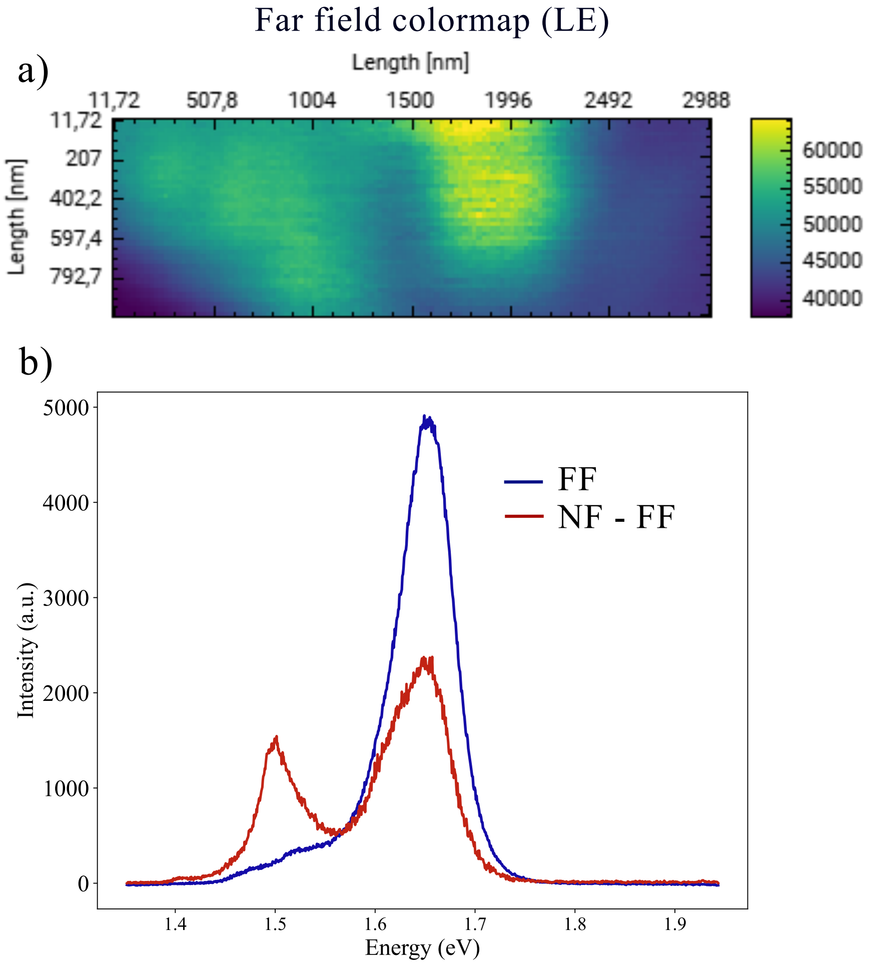

SI Figure 9 | Far-field for step-like platform. (a) Far-field colormap of the step-like platform shown in Fig. 6. (b) Representative spectra comparing the far-field signal with the near-field–far-field subtraction.

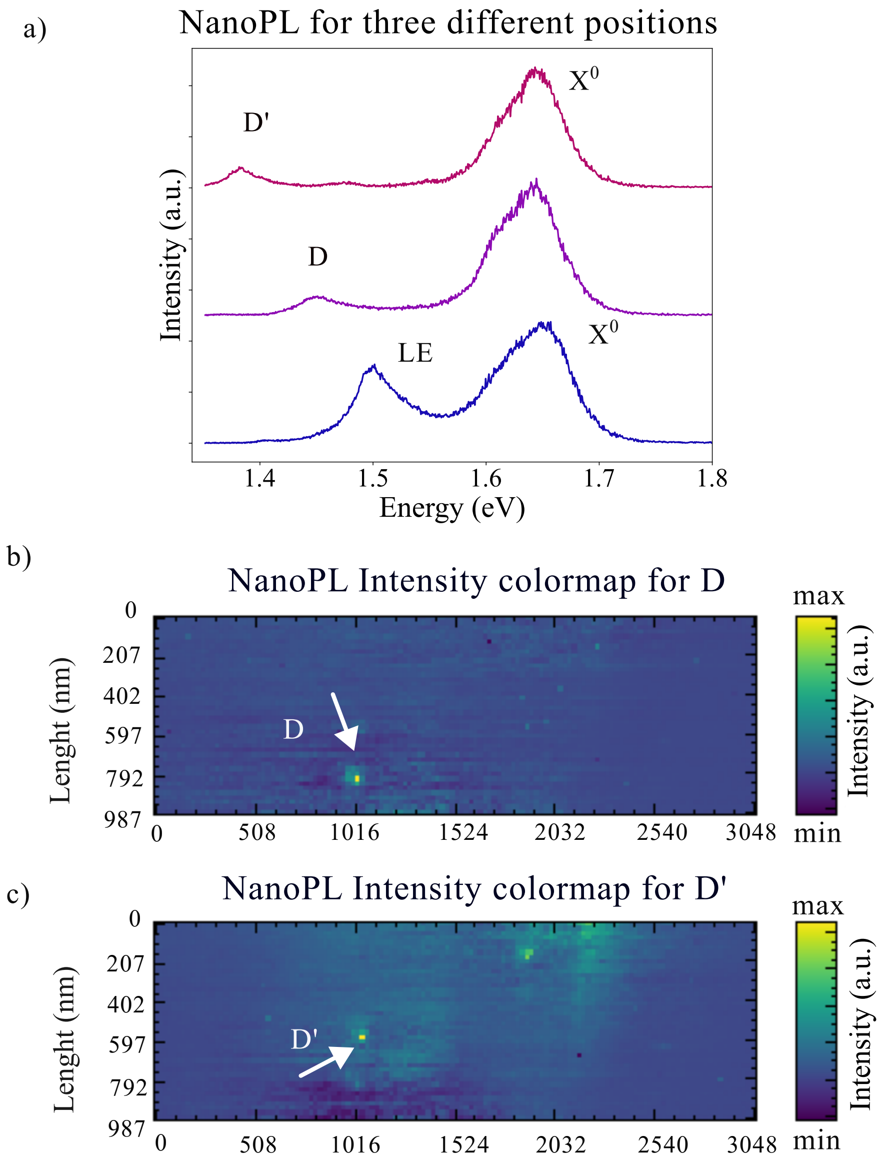

SI Figure 10 |Lower energy defect emissions. (a) NanoPL spectra for three different positions highlighting the LE and two other lower energy and intensity emissions. (b) NanoPL intensity colormap for D and D' energies showing a single pixel emission.
